# Nonconventional driving force for selective oxidative C–C coupling reaction due to concurrent and curious formation of Ag^0^

**DOI:** 10.1038/s41598-021-81020-1

**Published:** 2021-01-15

**Authors:** Khushboo Bhanderi, Prasanna S. Ghalsasi, Katsuya Inoue

**Affiliations:** 1 Department of Chemistry, Faculty of Science, The Maharaja Sayajirao University of Baroda, Vadodara, Gujarat 390002 India; 2 Department of Chemistry, Graduate School of Science and Chirality Research Center (CResCent), Hiroshima University, 1-3-1, Kagamiyama, Higashi Hiroshima, Hiroshima, 739-8526 Japan

**Keywords:** Catalysis, Organic chemistry, Surface chemistry, Chemical synthesis

## Abstract

Is it possible to ‘explore’ metal’s intrinsic property—a cohesive interaction—which naturally transform M^0^ into an aggregate or a particle or film for driving oxidative C–C bond formation? With this intention, reduction of [Ag(NH_3_)_2_]^+^ to Ag^0^ with concurrent oxidation of different phenols/naphthols to biphenyls was undertaken. The work is originated during careful observation of an undergraduate experiment—Tollens’ test—where silver mirror film deposition takes place on the walls of borosilicate glass test tube. When the same reaction was carried out in polypropylene (plastic-Eppendorf) tube, we observed aggregation of Ag^0^ leading to floating Ag-particles but not silver film deposition. This prompted us to carry out challenging cross-coupling reaction by ONLY changing the surface of the reaction flask from glass to plastic to silicones. To our surprise, we observed good selective oxidative homo-coupling on Teflon surface while cross-coupling in Eppendorf vial. Thus, we propose that the formation of biphenyl is driven by the macroscopic growth of Ag^0^ into [Ag-particle] orchestrated by Ag…Ag cohesive interaction. To validate results, experiments were also performed on gram scale. More importantly, oxidation of β-naphthol carried out in quartz (chiral) tube which yielded slight enantioselective excess of BINOL. Details are discussed.

## Introduction

### Role of [Ag(NH_3_)_2_]^+^ for oxidative BINOL formation

Bernard Tollens (1841–1918) realized the potential of silver complex, [Ag(NH_3_)_2_]^+^, to oxidize aldehyde group to carboxyl group, with concomitant reduction of Ag^+^ to silver mirror/film (Ag^0^), on the inner walls of the test tube^1,2^. The potential of this reaction has been exploited only in chemistry education—as a test to distinguish aldehydes from ketones—mainly due to its ease and wide applicability on variety of aldehydes. Interestingly, oxidation potential (+ 0.34 eV) for conversion of [Ag(NH_3_)_2_]^+^ to Ag^0^ matches well to that of direct oxidative C–C coupling between phenols^3,4^. In this observation we saw a potential in conducting a pair of redox reaction involving Ag^+^ to Ag^0^ and phenol to bisphenol as coupled reactions. This prompted us to carry out Tollens' test on *β*-naphthol, a common phenol employed for bisphenol formation, as the product bisnaphthol itself finds importance in asymmetric synthesis and material science^5,6^. The reaction gave a positive result, meaning formation of a silver mirror on the walls of test tube. The product, 1,1′-bi-2-naphthol (BINOL), in this reaction can be isolated by simple decantation! easy separation from the deposited silver film.

To the best of our knowledge, this is the first report demonstrating the use of silver complex, [Ag(NH_3_)_2_]^+^, for a direct carbon–carbon bond oxidative coupling reaction^7^.

### [Ag(NH_3_)_2_]^+^ for symmetric biphenyl formation

**Figure 1:**
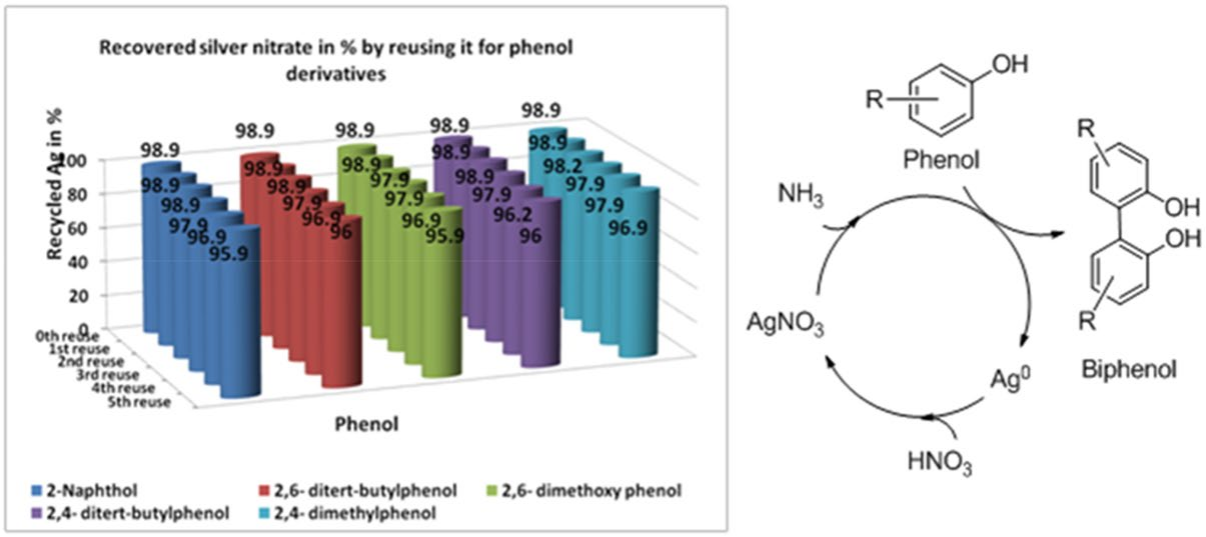
A graph of recovered silver nitrate (%) after each C–C oxidative coupling reaction between phenol or naphthol derivatives. Schematic recovery of silver is shown on right hand side.

Experimentally the reaction of *β*-naphthol to BINOL conversion was carried out similar to Tollens’ test with two distinct changes (1) aldehyde is replaced by phenol and (2) all chemicals were taken in exact 1:1 stoichiometric quantities as per the redox equation (as per general procedure given below and Fig. 1). We would like to mention here, that competition reaction between derivatives of phenol and benzaldehyde, in presence of NaOH yielded oxidation of aldehydes while, in absence of NaOH paved way for C–C oxidative coupling reaction. We also found necessary of ‘oxygen’ atmosphere than pure nitrogen, air, and carbon dioxide atmosphere.

After optimizing experimental conditions, the reaction was performed on derivatives of naphthol and phenol (Table 1). Complete characterization of all the products is given below. We observed formation of *ortho-ortho* homo-coupling during biphenyl formation, when *ortho*-positions were substituted by *tert*-butyl/methoxy/methyl groups, then *para-para* homo-coupling. Addition of quantitative amount of NaOH in the *para-para* coupling reaction led to substantial quinone formation. The gram scale reaction of phenol 2a resulted in 91% 2aa formation, isolated column purified, which shows feasibility of the reaction in larger scale. (as shown in experimental part) One can realize reaction displays novel catalytic behavior, where reaction proceeds homogeneously for ensuing complete conversion of reactants and then transforms to heterogeneous condition for easy recovery of the products.

**Table 1:** Oxidative C–C homo-coupling of substituted phenols. Isolated yield of column purified product is reported.

Substrate	Product	Reagent	Solvent	Temp	Time	Yield (%)
		AgNO_3_ + NH_3_	Ethanol	30 °C	44 h	90
		AgNO_3_ + NaOH + NH_3_	Ethanol	30 °C	44 h	87
		AgNO_3_ + NH_3_	Ethanol	70 °C	25 min	73
		AgNO_3_ + NH_3_	Ethanol	30 °C	1 h	95
		AgNO_3_ + NaOH + NH_3_	Ethanol	30 °C	1 h	83
		AgNO_3_ + NH_3_	Ethanol	70 °C	5 min	83
		AgNO_3_ + NH_3_	Ethanol	30 °C	10 min	70
		AgNO_3_ + NaOH + NH_3_	Ethanol	50 °C	5 min	66
		AgNO_3_ + NH_3_	Ethanol	30 °C	30 min	85
		AgNO_3_ + NaOH + NH_3_	Ethanol	30 °C	30 min	83
		AgNO_3_ + NH_3_	Ethanol	50 °C	2 min	79
		AgNO_3_ + NH_3_	Ethanol	30 °C	5 min	72
		AgNO_3_ + NaOH + NH_3_	Ethanol	70 °C	5 min	25
		AgNO_3_ + NH_3_	ethanol	30 °C	5 min	74
		AgNO_3_ + NaOH + NH_3_	ethanol	30 °C	10 min	55
		AgNO_3_ + NH_3_	Ethanol	30 °C	72 h	62

Typical reaction parameters are—AgNO_3_ (0.2 mmol), liquor ammonia (0.2 ml), substrate (0.2 mmol), absolute ethanol (0.5 ml).

### Recycling of generated silver film

The deposited silver mirror film can be completely recovered (about 99% after 1st use) and reused by dissolving it with the addition of HNO_3_ (10 N). Recycling of the ‘silver mirror’ was successfully carried out up to 5 cycles with more than 95% recyclability (presented in Fig. 1). Quantification of the amount of deposited silver using *conductometric* analysis was also carried out by titrating silver nitrate with standard KCl solution.

### Mechanism for Regio-selectivity: radical–radical or radical-anion coupling

**Figure 2:**
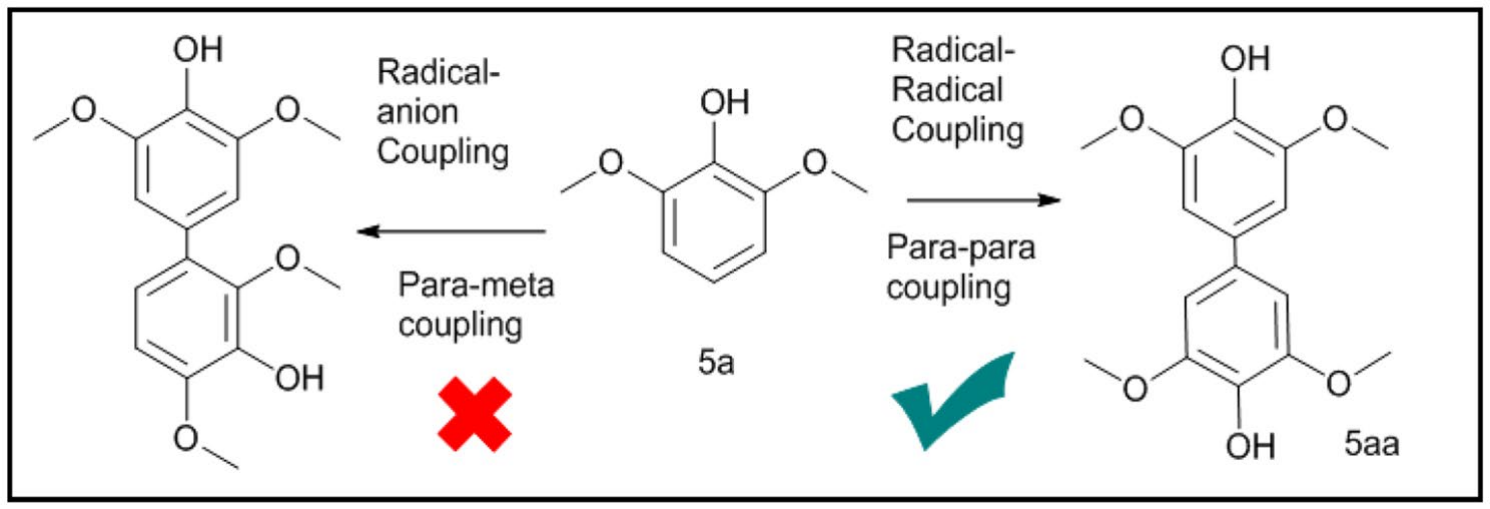
Selective homo-coupling of 2,6-di-methoxy phenol (5a): (right side) *para-para* coupled product (5aa) due to radical–radical coupling; (left side) *para-meta* coupled product due to radical-anion coupling reaction mechanism.

In literature oxidative homo-coupling reaction of 2,6-dimethoxyphenol (5a) has shown formation of both *para-meta* and *para-para* coupled products (as shown in Fig. 2)^3,8–12^. Former is due to the preferred radical-anion coupling, while the later (5aa) is due to radical–radical coupling (outer sphere homolytic coupling) reaction pathway as shown in Fig. 2. Formation of *para-meta* coupled product happens in presence of hexaflouroisopropanol (HFIP) solvent, where hydrogen bonding (around phenolic hydroxide) helps in increasing nucleophilic character at the *ortho*-position of methoxy group^4,13^. We want to point out here that, when the reaction of 2-naphthol was carried out in the presence of equimolar 2,6-di-tert-butyl-4-methyl phenol, it showed retention of large amount of unreacted reactant, indicating radical quenching of intermediate state^14,15^. This is in accordance with the formation of only *para-para* product (5aa), a radical–radical coupling pathway, as reported in literature.

### Oxidative C–C bond formation: homo- or cross-coupling

Oxidative coupling reaction between two different phenols normally leads to major homo-coupled product formation. With this background, when two phenols 2,6-dimethoxyphenol (5a) and 2,6-di-*tert*-butylphenol (3a), on treatment with [Ag(NH_3_)_2_]^+^ yielded mixture of both cross-coupled and homo-coupled product (as shown in Table 2 entry 6). Here, we observed, cross-coupling reaction completes faster (in less than 5 min) than the respective homo-coupling reaction (in more than 10 min), although both cases ended in clear silver mirror formation.

**Table 2:** Cross coupling between 3a and 5a in different types of containers.

No	Surface	% yield of cross coupled products on repeated experiments	Cross coupled product yield in %	Homo coupled products yield in %		Coupling selectivity	Time in min	Nature of Silver formed in the reaction
			c	3aa	5aa			
1	Polyethylene	70–80	73	25	25	Cross	4–5	Fine particles
2	Polypropylene	60–65	62	25	25	Cross	5–6	Particles
3	Durasil glass	50–60	51	35	44	Cross	5–7	Film
4	Quartz	40–45	43	45	56	–	10–15	Film + particles
5	White marble	30–35	35	50	56	–	5–10	Film + particles
6	Borosilicate	30–35	35	60	62	–	4–5	Film
7	Stainless steel	25–30	32	60	62	–	10–15	Particles
8	Teflon	20–25	22	70	75	Homo	5–7	Film + particles

Time required for complete conversion, and % of isolated yield (after column) are reported. Range is given for cross-coupling products formed in the total yield in repeated experiments. (c and 3aa yields were calculate on the basis of 3a, 5aa yield was calculated on the basis of 5a).

Due to clean isolation of products, short time of the reaction, and beautiful mirror formation at the end of the reaction helped us to pose three curious questions, which are discussed below.

### Three sets of curious experiments: hypothesis development

#### Experiment 1:

Why to carry out reaction in boro-silicate glass tubes why not in plastic (polypropylene or polyethylene) container?

**Figure 3:**
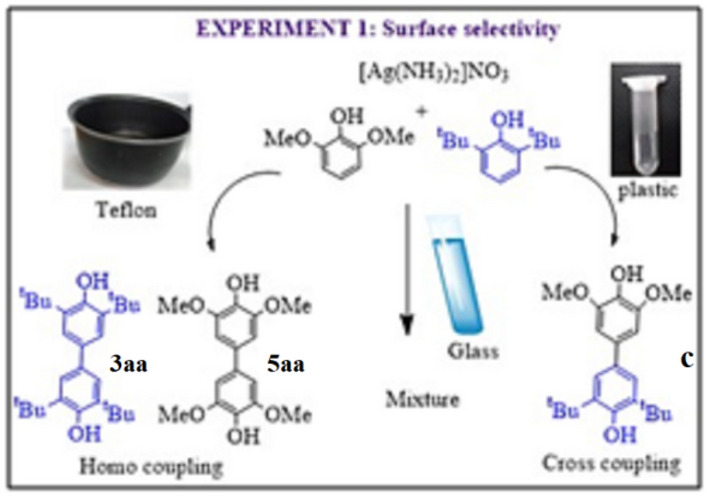
Two distinct Surfaces of a container directing C–C oxidative coupling between 2,6-dimethoxyphenol and 2,6-di-*tert*-butylphenol to major homo-coupling product (Teflon TM surface and major cross-coupling product (plastic (polypropylene and polyethylene)—Eppendorf tube). Reaction carried out in borosilicate glass tube resulted into the mixture of both these products.

Oxidative coupling reaction between 2,6-dimethoxyphenol and 2,6-di-*tert*-butylphenol with [Ag(NH_3_)_2_]^+^ resulted in silver mirror film formation on the walls of borosilicate glass tube along with the mixture of cross- and homo-coupled products. Interestingly, same reaction (without changing experimental conditions) when carried out in a plastic vial (eppendorf tube of polypropylene), furnished floating silver particles in the solution instead of silver mirror formation. This observation prompted us to pose a question, is the change in the growth of Silver mirror formation to silver particle has any influence on C–C oxidized product? We posed this question because mechanistically origin of formation of Ag^0^ and C–C coupling, exchange of electron, is the same. To answer this question, we designed a few more experiments, basically without changing any experimental condition, reactions were performed on marble, stainless steel, TeflonTM, plastic-polypropylene, plastic-polyethylene, durasil glass, Borosilicate glass and quartz surface containing vessels. Complete results are shown in Fig. 3 and Table 2.

Although all results shown in Table 2 are interesting and may lead to further set of up experiments, but two clear striking outcomes are (1) plastic (Polyethylene- Eppendorf tube) container yielded major cross-coupled product, while (2) Teflon surface results in major homo-coupled product. All the reactions were repeated minimum 3 times, and isolated yield is reported after column chromatography. Reactions were also carried out on gram scale to confirm validity of the observations. When compared these results with literature, similar selectivity for cross and homo-coupled products formation was normally observed only after employing totally different experimental conditions, such as catalyst, concentration of reagents, solvent, and temperature etc^8–11,13,16,17^. We would like to mention here that present set of reactions carried were also carried out in different sizes of the containers, but did not show any significant improvement of overall selectivity of the product formation. (shown in SI-32).

Traditionally, in literature, minimization of homo-coupling is achieved, with limited success, by employing following strategies: (a) choosing appropriate phenols: Phenols with difference of more than 0.25 eV in the oxidation potential are normally reacted where the least oxidizable phenol is taken in large excess^3,9^ (b) use of *dimeric* or designer *ligand* metal complexes: Metal complexes of Cu(II), Fe(III), V(V), Cr(III) are used as catalysts (0.5 to 20 mol% loading) to increase intra-molecular coupling through cooperative (cluster) model^18^, (c) Use of *sterically* hindered phenols: help in restricting free rotation with prominent *nucleophilic* attack and thus resulting *regio*-isomeric product formation^19,20^, (d) Use of fluorinated solvent: to generate strong hydrogen bonded assemblies amongst the most redox active phenol derivative and then *nucleophilic* attack by the other phenol in micro-heterogeneous environment^8–10^. Basically most of the strategies in the literature, to the best of our knowledge, control molecular interactions to favor radical-anion interaction or radical–radical and/or radical *cation-*interactions, for the selectivity^4,17^. That is why in all these efforts the role of metal remained restricted to two functions, one to ‘exchange’ an electron and second to supply surface for two interacting phenols. That is why significant selectivity was also observed under metal free conditions^17,19,21^.

### Understanding silver mirror film as an aggregation of Ag^0^: development of hypothesis

In this reaction, formation of Silver metal (Ag^0^) can act as an epicenter where not only electron-exchange takes place but also concurrent oxidized product (consider here case of BINOL formation) formation takes place. That means, at intermediate formation is interlinked with the stability of both biphenyls, oxidized product, and concurrently reduced Ag^0^, and may thus growth of later may influence ‘topology’ of the former. With this understanding, one can decipher the formation of silver film from Ag^0^ in following three simple equations. Here, equation 1 represents ‘molecular’ level interaction between 2-naphthol and [Ag(NH_3_)_2_]^+^, typically a key and ONLY equation to represent redox behavior in the literature. Role of concurrent growth of Ag^0^ into ‘macroscopic’ level silver film formation remained unnoticed in the reaction progress. That is why we made an attempt to write eqsuations 2 and 3, where growth of Ag^0^ is initially driven by cohesive forces and latter by adhesive interactions with the surface, respectively^22^. Interestingly, reactions taking place in equaions 2 and 3 are optically visible, intermittent-short lived- dark brown precipitate formation and clear solution formation. These observations play significant role in understanding silver nano-particle generation.
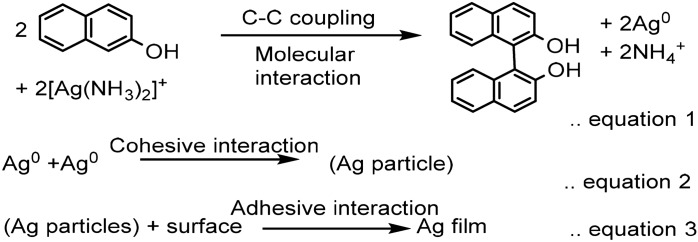


Interdependency between three equations can be realized only after considering reversible equilibrium condition between them. Literature do site presence of such reversible equilibrium behavior between equations 1 and 2^23–27^ (case-a) and equations 2 and 3 (case-b)^28–40^. This is first report which correlate relationship between equations 1 and 3, and highlight importance of cohesive interaction between metal for driving selectivity in molecular reaction.

Two most probable pathways for the present experimentally observed selectivity in homo and cross-coupling are, (in accordance with literature): (a) Monomeric complexes normally leads to major *homo*-coupled product while *dimeric* metal complexes major cross-coupled product^4,11^, that means in present case monomeric aggregation is favored under Teflon surface while dimeric aggregation in plastics; (b) formation of distinct sizes/shapes of Ag_particles_-Cohesive interaction under the influence of adhesive force by teflon and plastic may lead to distinct shape/size of silver cluster/agglomerate^8,26,35^, which are responsible for observed selectivity. Efforts to illustrate it using microscopic images were inconclusive, may be dominance of overgrowth of Ag-particle.

Interestingly, in literature clear observation of growth of Ag-intermediates in the form of aggregate/precipitate were reported but no attempts were made to correlate it with the observed selectivity in the oxidized product. Some related examples^41–46^; C–H/C–H functionalization^41^; oxidative furan formation^42^; C = N bond formation via C–H activation^43^; cross-coupling between C–H/P–H^44^; C–H arylation of primary aliphatic amines^45^; late stage fluorination^46^. All these reports may be undermining the role of Ag aggregates/precipitate formation (in large quantity) to the observed selectivity, interestingly highlighted importance and formation in the selective product formation, which needs detailed investigation.

#### Experiment 2:

Synthesis of BINOL in quartz, (chiral) surface. Can chiral surface enforces asymmetric synthesis or resolution of BINOL?

**Figure 4:**
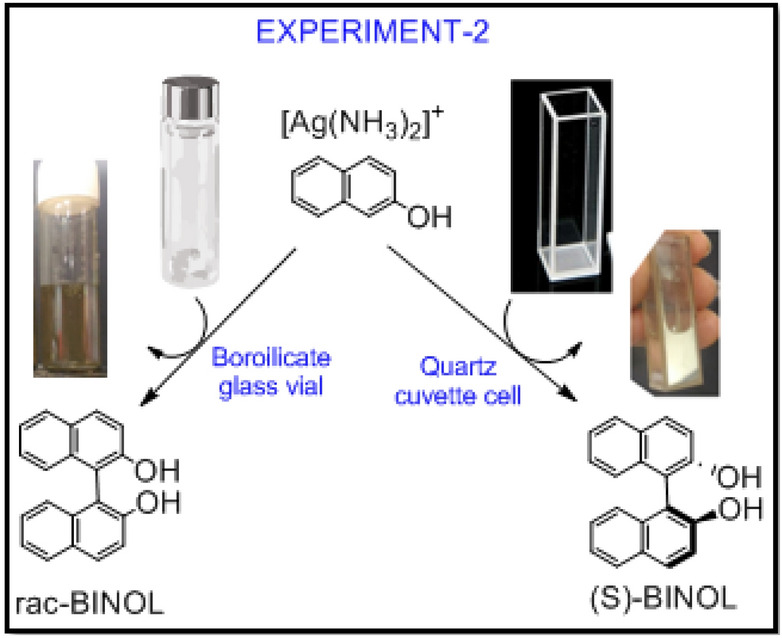
Role of silver film formation on the surface of the reaction vessel (-is this a chirality induction?): Reaction of [Ag(NH_3_)_2_]^+^ with 2-naphthol resulted in the S-BINOL (in slight but consistent enantiomeric excess) formation when reaction was carried out in quartz cuvette over borosilicate glass vial.

Reaction of [Ag(NH_3_)_2_]^+^ complex with 2-naphthol was carried out in quartz cuvette instead of borosilicate glass tube (Fig. 4). It took nearly 48-h for completion of the reaction, basically zero starting material. To our surprise, optical rotation showed 56% ee with 93% yield for this reaction mixture. The reaction was repeated just to observe reproducibility of the results, as shown in supporting information **SI-01A**. We did not make any efforts to increase %ee by optimizing reaction conditions or experimental parameters such as quality of quartz cuvette, surface area of adhesion, and more importantly shape-area correction.

It is normally accepted that the resolution and transformation of chiral molecules require some kind of chiral interactions, normally it is controlled by ‘sorption’, absorption/adsorption, with the surrounding^47–49^. Selectivity, although small, in present experiment can be realized only by assuming (a) complete reversible equilibrium reaction conditions between equations 1 to 3, and (b) assuming homochiral surface of quartz glass cuvette. The mechanism can be formulated for observing former mechanism: step 1—Chiral surface due to energy driven adhesive interaction induce or prefer generation of chiral Ag_particle_; step-2—cohesive interaction amongst Ag^0^ get driving force to aggregate in chiral manner; step-3—since Ag^0^ existence is linked with 2-naphthol and/or BINOL formation, latter also get transformed in the chiral generation. In short, in present case chirality from the surface is initially transferred to aggregation of silver particles and finally in the BINOL formation. That means, one may observe higher enantioselectivity, if the chiral crystal is employed. But this limitation prompted us to design and carry out third experiment.

#### Experiment 3:

Can aldehyde transfer its chirality to silver mirror film on borosilicate glass?

**Figure 5:**
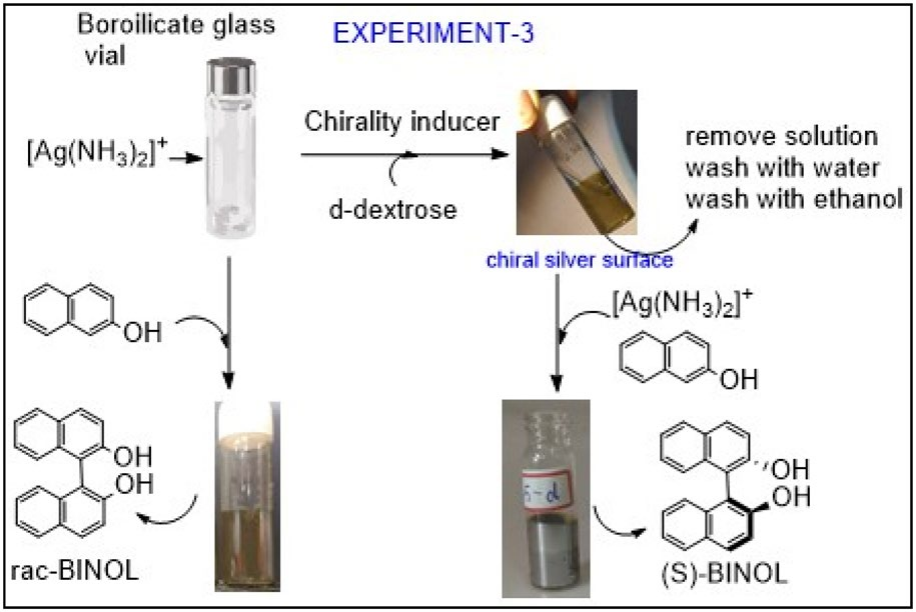
Attempted observation of chiral silver film formation with concurrent oxidation of d-dextrose. Instead of ‘characterization’ of silver mirror film in a borosilicate glass vial, it is directly tested for observing enantioselective BINOL formation. Although, observed selectivity is very weak but consistency in results and proposed mechanism provoke in depth investigation.

In continuation of the proposed mechanism, reversible equilibrium condition between equations 1 and 3, one can ‘imagine’ chiral silver film generation in the form of thin transparent mirror after [Ag(NH_3_)_2_]^+^ complex is reduced in presence of d-dextrose in borosilicate test tube. The extended logic in the present case is that since oxidized reactant/product is chiral then it may direct reduced product. One can employ detailed microscopic or high-end characterization techniques to probe chirality of this silver film^50–52^, but rather than that we choose to validate it by designing set of direct experiments. That is mixing the logic of experiment-2 and experiment 3. As shown in Fig. 5, oxidation of 2-naphthol in glass vial leads to racemic BINOL formation. Assuming the vial is coated by chiral silver film, obtained after oxidizing d-dextrose, when used for reaction between [Ag(NH_3_)_2_]^+^ and 2-naphthol, resulted in small enantiomeric excess of BINOL formation. Optical rotations did not show higher than 31% ee with 90–93% yield in the repeated experiments, but the all the experiments showed consistently around 5% ee (**SI-01B**). The observed optical rotation is exactly opposite to the starting material, d-dextrose, thus eliminating possibility of its adsorption or reminisce. We also would like to emphasis here that we never observed such results in borosilicate glass test tube, where by default racemic mixture is an outcome. During these experiments, we realized that optimizing concentrations of the reactants to get transparent silver mirror film is the most critical part, that means generating possibly monolayer of silver film on glass surface is key. All these experiments were carried out by taking utmost care and were reproducible in normal reaction conditions; we did not make an effort to optimize reaction conditions for higher ee.

Chiral thin film formation in gas phase conditions is well established, where chirality is induced by the use of organometallic chiral reagents^53^. Here chiral reagent, due to its low vapor pressure, do not take part in actual thin film formation but induce chirality. But to the best of our knowledge similar phenomena using liquid phase reactants is not cited in the literature. Although liquid phase film formation such as Langmuir Blodget (LB) film is also well established, but here chiral reagent remains in the lattice or film. On the other hand, generation of chiral nanoparticles using chiral organic molecules is well established, where chiral molecule stabilizes the chiral structure^53^.

We came across many references for in-situ aggregation or cluster formation before actually resulting in stereo or regio-selective product formation^54,55^. Few notable cases are octamer formation by serine during sublimation^56^, tetramer in Soai reaction^57^, or aggregation in crystallization of biominerals^22^. In present case similar aggregate formation, driven by the cohesive forces of Ag^0^, which are controlled by in-situ adhesive forces of Ag_particle_ to surface of the reaction vessel, cannot be ruled out.

So, the pattern of aggregation of Ag^0^, reducing component in the reaction, which is solely dependent on the surface adhesion dictate the geometric- as well as stereo-topology of the oxidized biphenyl formation. Traditionally, focus in organic chemistry remain on the stability of the oxidized organic intermediate for driving reaction in forward direction, and the role or fate of the reduced component remained unexplored. Thus, present manuscript makes an attempt to highlight this novel driving force for C–C coupling reaction.

In general, selectivity in organic transformation is known to get influenced by the size and shape of metal nanoparticles (as catalyst) and therefore most of the investigations in the literature are focused on detailed characterization of nanoparticles, which is not a topic of present work. Here, we realized potential of creating ‘in-situ’ different size and shapes of silver particles by changing surface of the reaction flask for achieving selective oxidative organic transformation. So, rather than investigating time and money on types of formation of silver particles, we focused on our attention on carrying out variety of reactions on different phenols with complete reproducibility and on gram scale synthesis. In present reaction, active metal complex, [Ag(NH_3_)_2_]^+^, reacts homogeneously with phenols (similar to homogeneous catalysis) and product results with the formation silver mirror film (similar to heterogeneous catalysis), for easy isolation of biphenyls.

In summary, with the help of simple and easily reproducible experiments we observed how in-situ formed Ag^0^ and its aggregation, help in driving selective oxidative carbon–carbon bond formation. The key to the observation is carefully writing of the reaction, silver mirror test or Tollens’ test, in three explicit reversible equations and correlating metals intrinsic cohesive interaction with the molecular level interactions. Present work has a potential to open up role of dynamics of this in-situ formed metal’s (silver) aggregation, non-interacting catalytic force, in driving selective oxidative organic transformation.

## Experimental section

### General

Silver nitrate was purchased commercially of 99.9999% purity. Potassium Chloride was purchased commercially of 99.999% of purity. All other reagents were of reagent grade quality, purchased commercially and used without further purification. TLC analyses were performed using silica gel aluminum plates 60 F254. The products were purified by column chromatography using Fluka chromatographic silica gel (40–60 µm). The products were identified with ^1^H NMR, ^13^C NMR spectroscopic and Mass spectrometric method shown in supporting information. NMR spectra were recorded on Advanced Bruker-400 (400 MHz) spectrophotometers; Chemical shifts are given in parts per million (ppm) relative to *tetra*methylsilane as an internal standard or to the residual solvent peak. Chemical shift multiplicity are shown as s = singlet, d = doublet, t = triplet, and m = *multiplet* and Coupling constants are given in Hertz (Hz). MS data was obtained from electron spray ionization mass spectral measurement (ESI–MS) or Gas chromatography mass spectrometry (GC–MS), which were performed using ESI-mass Applied Biosystem API 2000 mass spectrometer or Thermo scientific DSQ-II respectively. The optical purity of BINOL mixture was calculated from specific optical rotation data of mixture analyzed using Rudolph Research Autopol IV Automatic Polarimeter using sodium D-light shown in supporting information. The specific optical rotation of BINOL obtained in absolute alcohol (R)-( +)-BINOL and (S)-( −)-BINOL are + 30.0° and − 30.0° degree cm^2^ g^−1^ (C 0.3, at 28 °C) respectively.

### General procedure for C–C coupling

Silver nitrate (0.0340 g, 0.2 mmol) was dissolved into liq. Ammonia (~ 0.2 ml) using magnetic stirrer bar in a test tube to prepare Silver-ammine complex solution. The solution of phenol (0.2 mmol in 1.0 ml in absolute ethanol) was added into the silver-ammonia complex solution. The mixture was either stirred for stipulated time and at particular temperature on oil bath as shown in Table 1. All solution and organics were separated from crude metallic silver and organic phase were extracted with organic solvent such as ethyl acetate and dichloromethane and dried over Na_2_SO_4._ The volatiles were removed under reduce pressure and the crude was purified using column chromatography on silica gel, affording the coupling product.

Note: Silver ammine complex with sodium hydroxide was prepared by addition of 0.2 ml 0.1 N Sodium hydroxide solution in silver nitrate (0.0340 g, 0.2 mmol) followed by addition of liq. Ammonia (~ 0.2 ml).

### Procedure for gram scale C–C coupling reaction of phenol 2a

Silver nitrate (0.823gm, 4.85 mmol) was taken in a test tube then dissolved into 3.0 ml liq. Ammonia using magnetic stirrer bar in a round bottom flask. The solution of phenol 2a (1.000gm, 4.84 mmol in 3.0 ml in absolute ethanol) was added into the silver-ammonia complex solution. The mixture was either stirred for 60 min and at 30 °C temperature. The organics were separated and extracted with ethyl acetate and dried over Na_2_SO_4_ and evaporated. The crude was purified using column chromatography on silica gel. Product 2aa was obtained as a solid with 91% yield.

### Procedure for competition reaction of C–C coupling of phenol and oxidation of aldehyde

0.3 ml 0.1 N Sodium hydroxide Solution was added to the silver nitrate (0.051 g, 0.3 mmol), followed by addition of liq. ammonia (0.3 ml) solution to prepare silver ammine complex. The solution of 4-nitrobenzaldehyde (0.015 g, 0.1 mmol) and 2-Naphthol (0.014 g, 0.1 mmol) in absolute ethanol (2.0 ml) was added to the silver-ammonia complex solution. The mixture was stirred heated on an oil bath using a magnetic stirrer for 1 h. The crude was analyzed using TLC.

Note: This reaction also carried out using silver ammine complex prepared in absence of sodium hydroxide by dissolving silver nitrate (0.051 g, 0.3 mmol) in liquor ammonia (0.3 ml) solution.

BINOL formation only observed when reaction is carried out in absence of sodium hydroxide whereas BINOL along with 4-nitrobenzoicacid formation was observed when reaction is carried out using silver ammine complex prepared by addition of sodium hydroxide.

### General procedure for catalytic reuse of reagent

Silver nitrate (0.0340 g, 0.2 mmol) was dissolved into a liq. Ammonia (0.2 ml) in a test tube to prepare Silver-ammonia complex solution. The solution of phenol (0.2 mmol in 1.0 ml in absolute ethanol) was added into the silver-ammonia complex solution. The mixture was stirred at 30 °C or 70 °C temperature until complete consumption of reagent.

The organic phase was extracted with organic solvent such as ethyl acetate and dichloromethane from crude metallic silver. The remaining silver was washed using organic solvents followed by water and treated with minimum amount of nitric acid (10 N). The resultant silver nitrate was treated with ammonia until it become basic and used directly for further reaction using method shown above.

The recovered reagent was quantified by counductometric titration of recovered silver nitrate with standard potassium chloride solution. Six reactions were kept together and the reagents were reused 5 time and after every cycle one of them is used for titration.

### General procedure for cross coupling of phenol derivatives

Ammonical silver nitrate complex was prepared by dissolving silver nitrate (0.034 g, 0.2 mmol) into liquor ammonia solution (0.2 ml). 2,6-dimethoxy phenol (0.016 g, 0.1 mmol) and 2,6-di-*tert*-butylphenol (0.021 g, 0.1 mmol) was dissolved in absolute ethanol (0.5 ml) and added to vessel (borosilicate). The silver-ammonia complex solution was added into the solution of phenol at 30 °C and kept for 1 h after mixing by shaking the vessel for few seconds. The product was extracted from reaction mixture using DCM and ethyl acetate solvent (2–5 ml of each in 0.5 ml fraction). The extract was dried and residue was purified using column chromatography on silica gel using ethyl acetate in petroleum ether.

Note: for experiment 1 vessel used for reaction are made up of different material (such as polyethylene, polypropylene, durasil glass, quartz, white marble, borosilicate, stainless steel, teflon) as shown in Fig. 3 and Table 2.

### Procedure for C–C coupling of phenol 5a in presence in fluorinated solvent

Silver nitrate (0.0340 g, 0.2 mmol) was dissolved into liq. Ammonia (~ 0.2 ml) using magnetic stirrer bar in a test tube to prepare Silver-ammine complex solution. The solution of 2,6-di-methoxy-phenol (0.2 mmol in 1.0 ml in HFIP) was added into the silver-ammine complex solution. The mixture was either stirred for 3 h at 30 °C temperature. All solution and organics were separated from crude metallic silver and organic phase were extracted with organic solvent such as ethyl acetate and dichloromethane_._ The volatiles were removed under reduce pressure and the crude was purified using column chromatography on silica gel. The product 5aa was only obtained.

### General procedure for C–C coupling of 2-naphthol

Silver nitrate (0.043 g, 0.25 mmol) was dissolved in 1.0 ml liquor ammonia into the borosilicate glass tube. After 5 min 2-naphthol (0.036 g, 0.25 mmol) in 0.5 ml absolute ethanol was added and mixed by shaking the solution. This solution was kept in dark at 28–30 °C for 48 h. Resulted BINOL was separated and isolated.

### General procedure for experiment 2

Quartz vial of 3.5 ml capacity having 1 cm * 1 cm * 3.5 cm dimensions was used to carry out this reaction. Silver nitrate (0.043 g, 0.25 mmol) was dissolved in 1.0 ml liquor ammonia into the quartz tube. After 5 min 2-naphthol (0.036 g, 0.25 mmol) in 0.5 ml absolute ethanol was added and mixed by shaking the solution. This solution was kept in dark at 28–30 °C for 48 h. Resulted BINOL was separated and isolated. The specific optical rotation of resultant BINOL mixture was analyzed using polarimetry and optical purity was calculated in the form of enantiomeric excess.

### General procedure for experiment 3

1.0 ml of 0.01 N Silver nitrate solution was added to the oven dried borosilicate glass vial (cylindrical shape having 1 cm diameter and 5 ml capacity). After 1 min 0.1 ml of 0.10 N sodium hydroxide solution was added into it using 1.0 ml syringe. After 1 min 0.15 ml ammonia solution (0.0025% w/v) added slowly using 1.0 ml syringe and mixed by shaking. After 5 min 0.5 ml 0.012 N glucose solution was added into the vial, using pipette. 0.5 ml water was added into it. This reaction mixture was kept in the dark at 28–30 °C for 1 h. Solution in the vial was discarded. Vial was washed with water thrice. Ethanol washing was also given and kept 1–2 h for drying at room temperature. This silver coated surface was used instead of quartz for further C–C coupling reaction of BINOL using same procedure given for experiment 2.

#### [1,1′-binaphthalene]-2,2′-diol (1aa) (BINOL)^23,58,59^:

^1^H NMR (400 MHz, CDCl_3_, 291 K): δ 5.09 (2H, Broad, –OH), 7.17 (2H, d, *J* = 8.0 Hz), 7.28–7.43 (6H, m), 7.92 (2H, d, *J* = 8.0 Hz), 8.02 (2H, d, *J* = 8.8 Hz); ^13^C NMR (100 MHz, CDCl_3_, 291 K) δ 110.8, 117.8, 124.1, 124.2, 127.5, 128.4, 129.4, 131.5, 133.4, 152.8; GCMS m/z: [M − 1]^+1^: 285.8 (molecular weight: 286.10 gm/mol).

#### 3,3′,5,5′-tetra-*tert*-butyl-[1,1′-biphenyl]-2,2′-diol (2aa)^60^:

^1^H NMR (400 MHz, CDCl_3_, 291 K) δ1.34 (18H, s), 1.47 (18H, s), 5.24 (2H, broad, –OH), 7.13 (2H, d, *J* = 2.4 Hz), 7.41 (2H, d, *J* = 2.4 Hz); ^13^C NMR (100 MHz, CDCl_3_, 291 K) δ 29.6, 31.6, 34.5, 35.2, 122.3, 124.8, 125.3, 136.2, 142.9, 149.8; ESI–MS m/z: [M]^+1^: 410.2 (molecular weight: 410.16 gm/mol).

#### 3,3′,5,5′-tetra-*tert*-butyl-[1,1′-biphenyl]-4,4′-diol (3aa)^60^:

^1^H NMR (400 MHz, CDCl_3_, 291 K) δ 1.38 (36H, s), 7.72(4H, s); ^13^C NMR (100 MHz, CDCl_3_, 291 K) δ 30.3, 34.4, 124.1, 133.9, 135.9, 152.8; GCMS m/z: [M]^+1^: 410.2 (molecular weight: 410.16 gm/mol).

#### 3,3′,5,5′-Tetra-*tert*-butyl-4,4′-diphenoquinone (3bb)^23^:

^1^H NMR (400 MHz, CDCl_3_, 291 K) δ 1.29 (36 H, s), 7.63 (4H, s); ^13^C NMR (100 MHz, CDCl_3_, 291 K) δ 30.34, 36.0, 126.0, 136.2, 150.5, 186.5; ESI–MS m/z: [M + 1]^+1^ 409.4 (molecular weight: 408.62 gm/mol).

#### 3,3′,5,5′-tetramethyl-[1,1′-biphenyl]-2,2′-diol (4aa)^58^:

^1^H NMR (400 MHz, CDCl_3_, 291 K) δ 2.29(12H, d, *J* = 0.8 Hz), 5.09 (2H, broad, OH), 6.88 (2H, d, *J* = 0.8 Hz), 7.02 (2H, d, *J* = 2.0 Hz); ^13^C NMR (100 MHz, CDCl_3_, 291 K) δ 16.2, 20.5, 122.1, 125.2, 128.5, 130.0, 132.0, 149.1; ESI–MS m/z: [M]^+1^: 242.3 (molecular weight: 242.32 gm/mol).

#### 3,3′,5,5′-tetramethoxy-[1,1′-biphenyl]-4,4′-diol (5aa)^23^:

^1^H NMR (400 MHz, DMSO-D_6_, 291 K) δ 3.84 (12H, s), 6.82 (4H, s), 8.33 (2H, broad, –OH); ^13^C NMR (100 MHz, DMSO-D_6_, 291 K) 56.7, 104.8, 131.8, 135.4, 148.7; ESI–MS m/z: [M + 1]^+1^: 307.1 (molecular weight: 306.31 gm/mol).

#### 3,3′,5,5′-tetramethoxy-4,4′-diphenoquinone (5bb)^59,61^:

^1^H NMR (400 MHz, CDCl_3_, 291 K) δ 3.84 (12H, s), 5.87 (4H, s); ^13^C NMR (100 MHz, CDCl_3_, 291 K) δ 56.5, 107.4, 157.3, 186.9; ESI–MS m/z: [M + 1]^+1^: 305.2 (molecular weight: 304.30 gm/mol).

#### 3,3′-di-*tert*-butyl-5,5′-dimethoxy-[1,1′-biphenyl]-2,2′-diol **(6aa)**^60,62^:

^1^H NMR (400 MHz, CDCl_3_, 291 K) δ 1.42 (18H, s), 3.77 (6H, s), 5.05 (2H, broad, –OH), 6.63 (2H, d, 2.8 Hz), 6.96 (2H, d, 2.8 Hz); ^13^C NMR (100 MHz, CDCl_3_, 291 K) δ 29.5, 29.7, 35.2, 55.7, 111.7, 115.3, 123.3, 138.9, 145.9, 153.2; ESI–MS m/z: [M]^+1^: 358.0 (molecular weight: 358.21 gm/mol).

#### [1,1′-binaphthalene]-2,2′,7,7′-tetraol (7aa)^23^:

^1^H NMR (400 MHz, DMSO-D_6_, 291 K) δ 6.26 (2H, d, *J* = 2.0 Hz), 6.75 (2H, dd, *J* = 2.4, 8.8 Hz), 7.04 (2H, d, *J* = 8.8 Hz), 7.66 (4H, m), 8.20 (2H, broad, –OH), 9.20 (2H, broad, –OH); ^13^C NMR (100 MHz, CDCl_3_, 291 K) δ 106.9, 113.1, 115.9, 116.2, 125.0, 130.7, 130.8, 136.8, 154.5, 156.6; GCMS m/z: [M − 1]^+1^: 317.4 (molecular weight: 318.32 gm/mol).

#### 3,5-di-*tert*-butyl-3′,5′-di-methoxy-[1,1′-biphenyl]-4,4′-diol (c)^63^:

^1^H NMR (400 MHz, CDCl_3_, 291 K) δ 1.39 (18H, s), 3.97 (6H, s), 7.00 (2H, s), 7.63 (2H, s); ^13^C NMR (100 MHz, CDCl_3_, 291 K) δ 29.3, 29.6, 29.7, 36.0, 55.8, 104.1, 125.6, 135.0, 150.0, 153.3; ESI–MS m/z: [M − 1]^+1^: 357.1 (molecular weight: 358.21 gm/mol).

## Supplementary Information

Supplementary Information.
