# Supplementary material for: Nonconventional driving force for selective oxidative C–C coupling reaction due to concurrent and curious formation of Ag0
Source: Sci Rep. 2021 Jan 15;11:1568. doi: 10.1038/s41598-021-81020-1 (PMC7811016; doi:10.1038/s41598-021-81020-1)
Supplement: Supplementary file 1 — Supplementary Information. [file 41598_2021_81020_MOESM1_ESM.doc]

**Supporting information for:**

Nonconventional Driving force for Selective Oxidative C-C Coupling reaction due to Concurrent and Curious formation of Ag^0^

Khushboo Bhanderi^1^, Prasanna S. Ghalsasi^*1,2^, Katsuya Inoue^2^

^1^Department of Chemistry, Faculty of Science, The Maharaja Sayajirao University of Baroda, Vadodara, Gujarat, India-390002. *Email: [prasanna.ghalsasi-chem@msubaroda.ac.in](mailto:prasanna.ghalsasi-chem@msubaroda.ac.in)

^2^Department of Chemistry, Graduate School of Science and Chirality Research Center (CResCent),

Table of Contents

SI-01 Optical purity of BINOL data and calculation

SI-02 ^1^H NMR of 1aa in CDCl_3_

SI-03 ^13^C NMR of 1aa in CDCl_3_

SI-04 ^1^H NMR of 2aa in CDCl_3_

SI-05 ^13^C NMR of 2aa in CDCl_3_

SI-06 ^1^H NMR of 3aa in CDCl_3_

SI-07 ^13^C NMR of 3aa in CDCl_3_

SI-08 ^1^H NMR of 3bb in CDCl_3_

SI-09 ^13^C NMR of 3bb in CDCl_3_

SI-10 ^1^H NMR of 4aa in CDCl_3_

SI-11 ^13^C NMR of 4aa in CDCl_3_

SI-12 ^1^H NMR of 5aa in DMSO-D_6_

SI-13 ^13^C NMR of 5aa in DMSO-D_6_

SI-14 ^1^H NMR of 5bb in CDCl_3_

SI-15 ^13^C NMR of 5bb in CDCl_3_

SI-16 ^1^H NMR of 6aa in CDCl_3_

SI-17 ^13^C NMR of 6aa in CDCl_3_

SI-18 ^1^H NMR of 7aa in DMSO-D_6_

SI-19 ^13^C NMR of 7aa in CDCl_3_

SI-20 ^1^H NMR of c in CDCl_3_

SI-21 ^13^C NMR of c in CDCl_3_

SI-22 to SI-31 Mass spectra

SI-32 Cross coupling reaction by varying size of vessel surface

**SI-01 Optical purity of BINOL data and calculation**

90-93% BINOL was obtained from C-C coupling reaction. The enantiomeric excess (ee) was calculated from measurement of SOR of BINOL.

All measurements were obtained using digital polarimeter with 0.5 dm path length and sodium D-light source and 0.3 w/v % g/ml concentration otherwise noted.

Optical purity or enantiomeric excess (ee) (in %) = [α]_obs_×100 /[α]_max_

= observed optical rotation of a sample×100/ specific optical rotation

Specific optical rotation (SOR) of S-BINOL and R-BINOL in absolute alcohol is -30.0 °.cm^2^.g^-1^ and +30.0 °.cm^2^.g^-1^ (C 0.3, at 28°C) respectively obtained experimentally.

A: Reaction of [Ag(NH_3_)_2_]^+^ complex with 2-naphthol in quartz cuvette : method 2

| **α in °** | **SOR of mixture**  **°.cm^2^.g^-1^** | **w/v**  **% (g/ml)** | **ee in %** |  | **Yield in %** |
| --- | --- | --- | --- | --- | --- |
| **-0.0252** | -16.8000 | 0.3 | 56.0 | S | 93% |
| **-0.0212** | -14.1333 | 0.3 | 47.1 | S | 92% |
| **-0.0098** | -6.5333 | 0.3 | 21.8 | S | 93% |
| **-0.0005** | -0.3556 | 0.3 | 1.18 | S | 93% |
| **-0.0012** | -0.8222 | 0.3 | 2.7 | S | 93% |

B: Synthesis of BINOL using silver film formed using d-dextrose : method 3

| **α in °** | **SOR of mixture**  **°.cm^2^.g^-1^** | **w/v**  **% (g/ml)** | **ee in %** |  | **Yield in %** |
| --- | --- | --- | --- | --- | --- |
| **-0.0139** | -9.2889 | 0.3 | 31.0 | S | 93% |
| **-0.0055** | -3.6889 | 0.3 | 12.3 | S | 93% |
| **-0.0005** | -0.3556 | 0.3 | 1.2 | S | 93% |
| **-0.0009** | -0.5778 | 0.3 | 1.9 | S | 93% |
| **-0.0033** | -2.2222 | 0.3 | 7.4 | S | 93% |
| **-0.0024** | -1.6222 | 0.3 | 5.4 | S | 93% |
| **-0.0029** | -1.9556 | 0.3 | 6.5 | S | 92% |
| **-0.0025** | -1.6889 | 0.3 | 5.6 | S | 92% |

**SI-02 ^1^H NMR of 1aa in CDCl_3_**

**
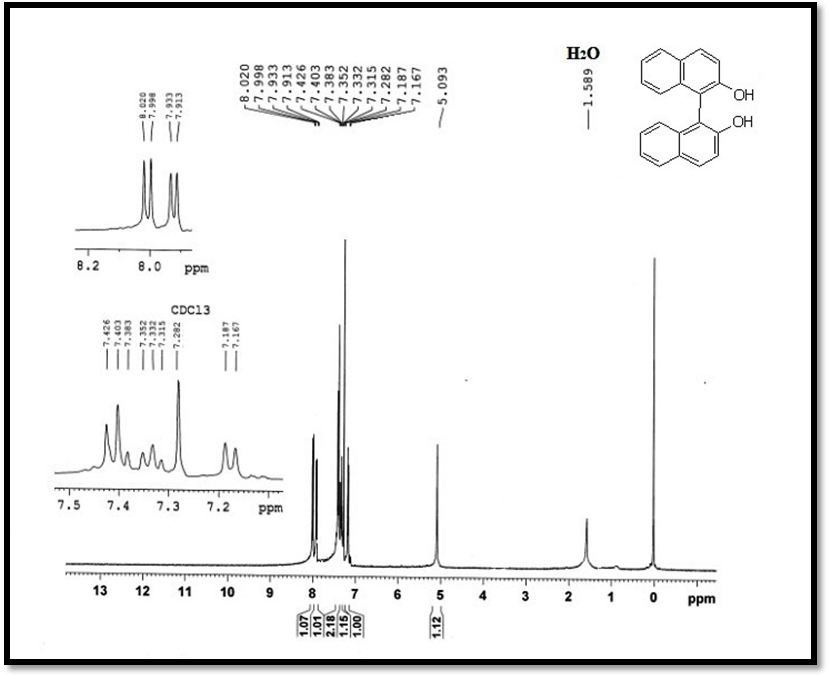
**

**SI-03 ^13^C NMR of 1aa in CDCl_3_**

**
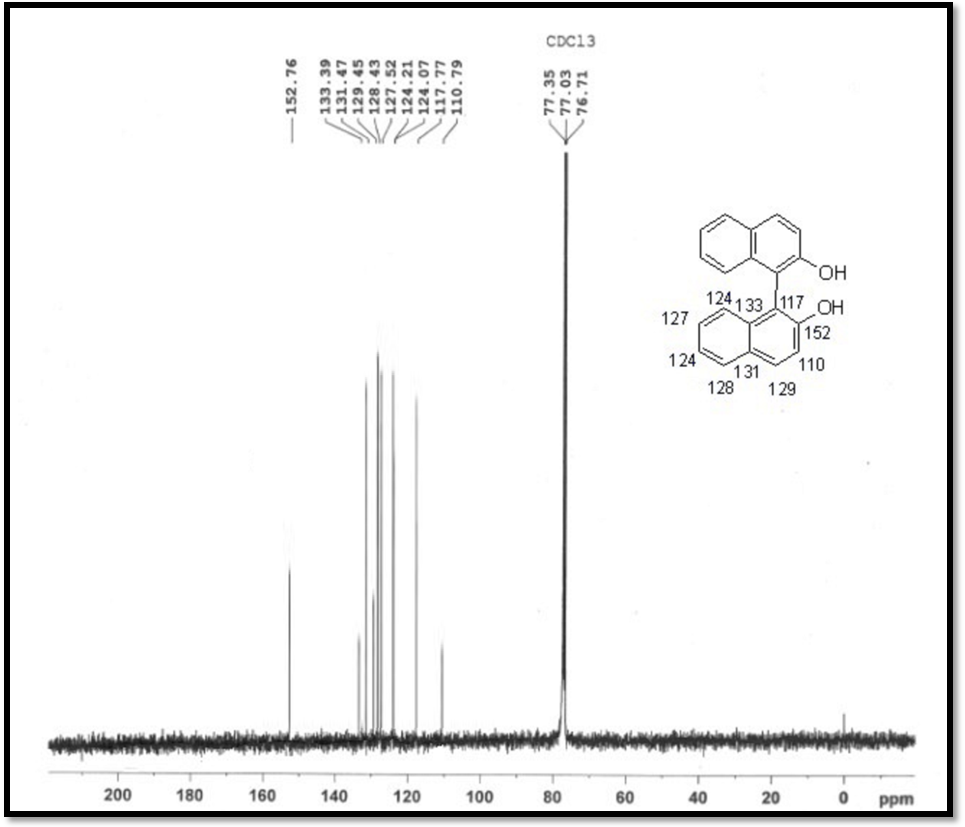
**

**SI-04 ^1^H NMR of 2aa in CDCl_3_**

**
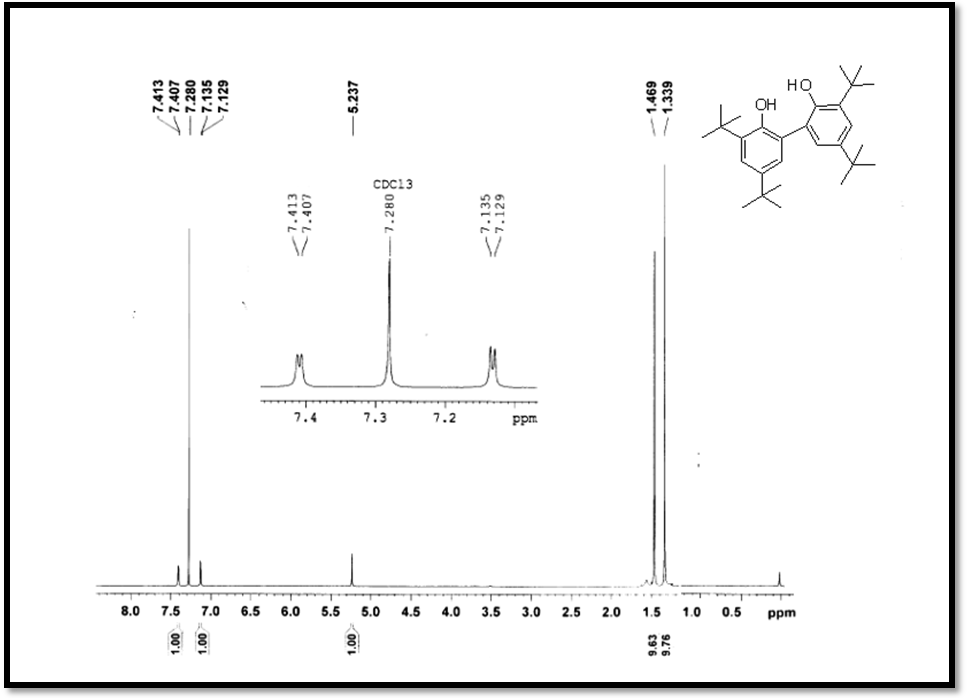
**

**SI-05 ^13^C NMR of 2aa in CDCl_3_**

**
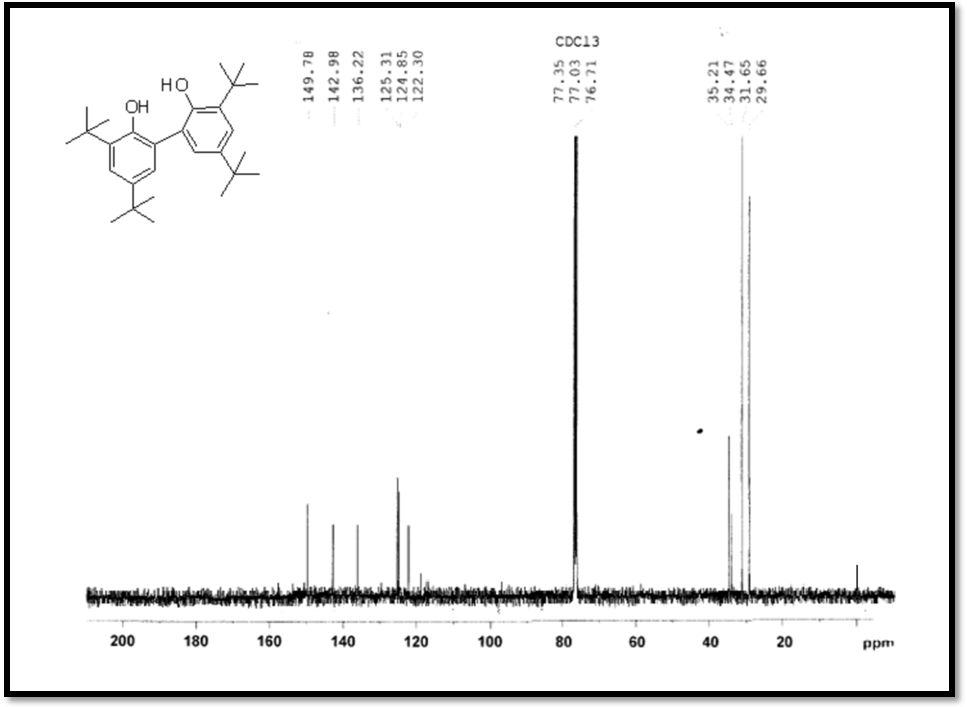
**

**SI-06 ^1^H NMR of 3aa in CDCl_3_**

**
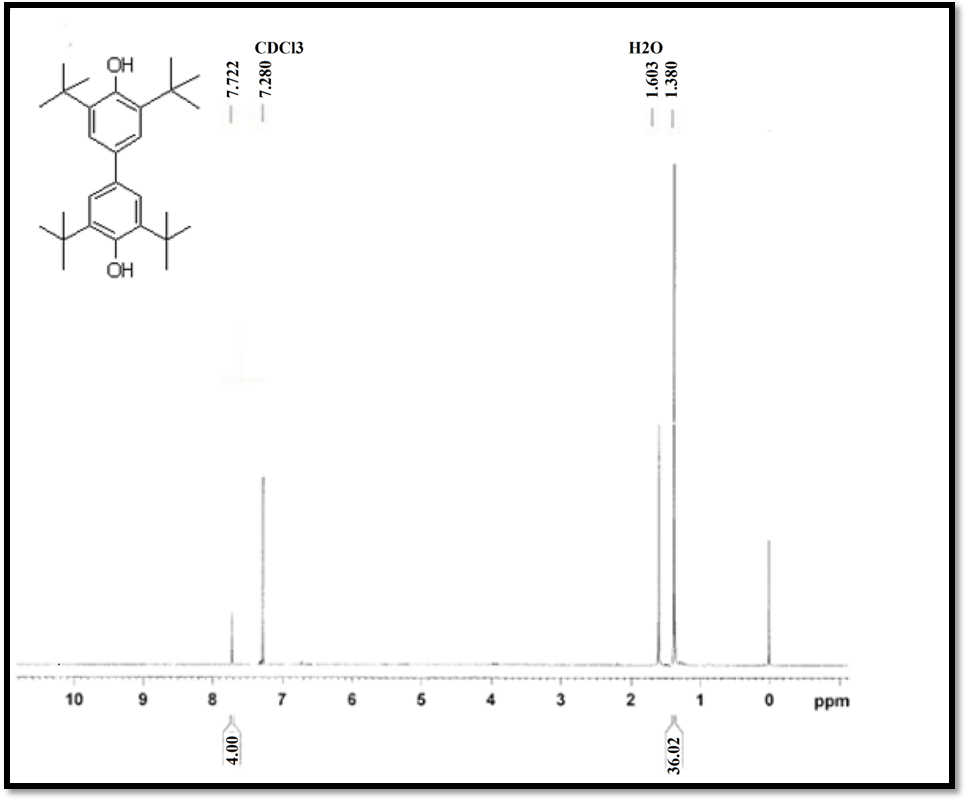
**

**SI-07 ^13^C NMR of 3aa in CDCl_3_**


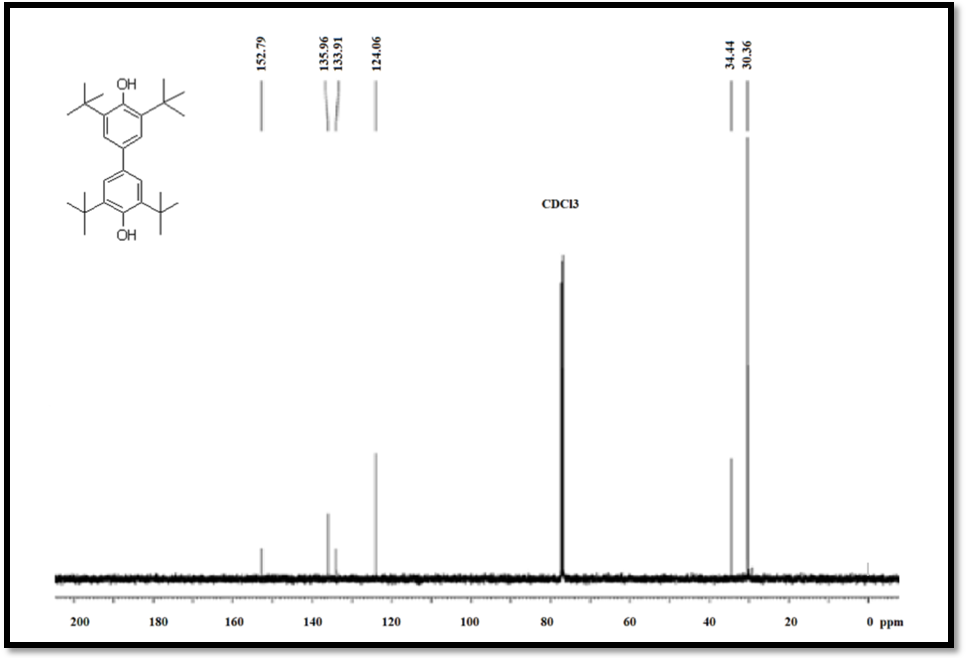


**SI-08 ^1^H NMR of 3bb in CDCl_3_**


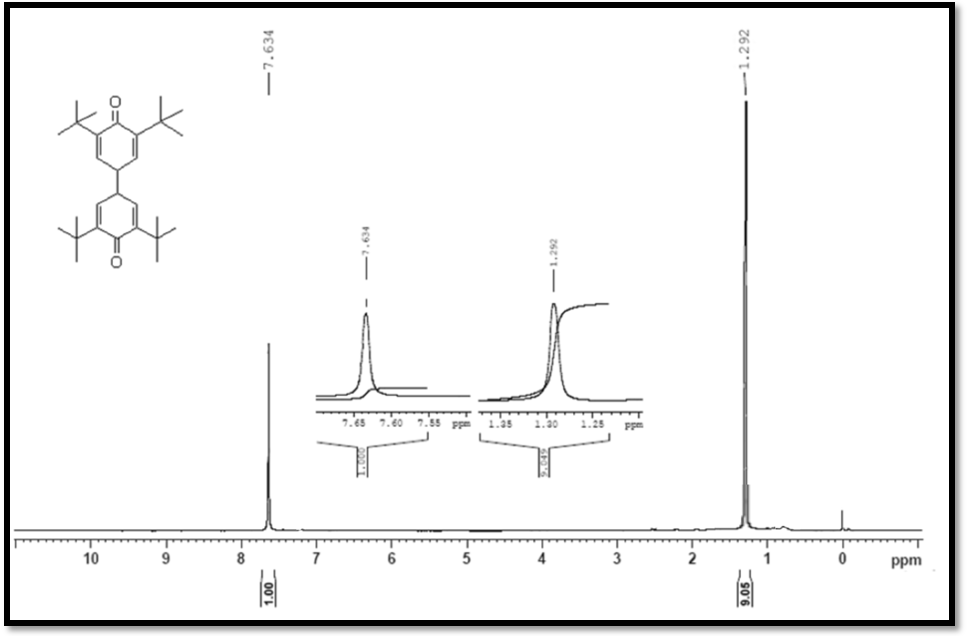


**SI-09 ^13^C NMR of 3bb in CDCl_3_**


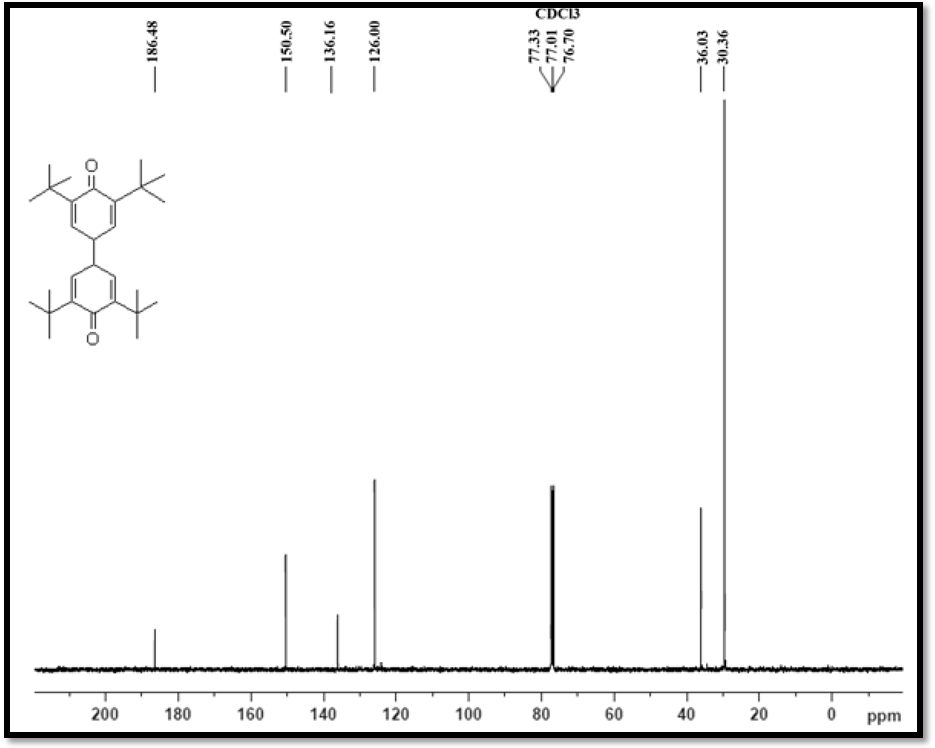


**SI-10 ^1^H NMR of 4aa in CDCl_3_**

**
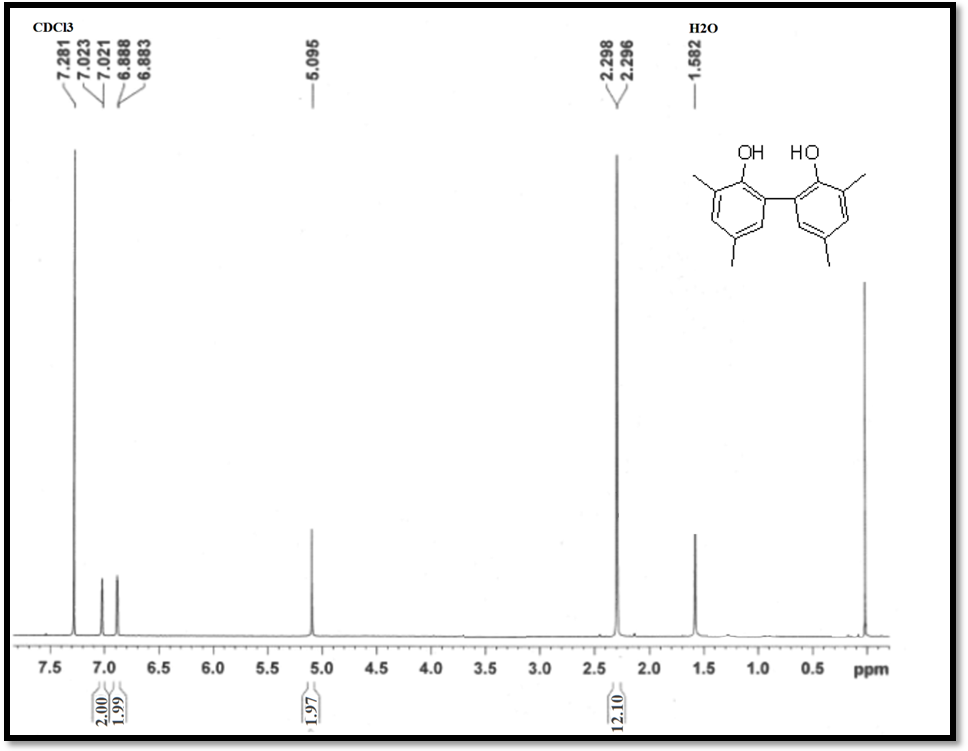
**

**SI-11 ^13^C NMR of 4aa in CDCl_3_**

**
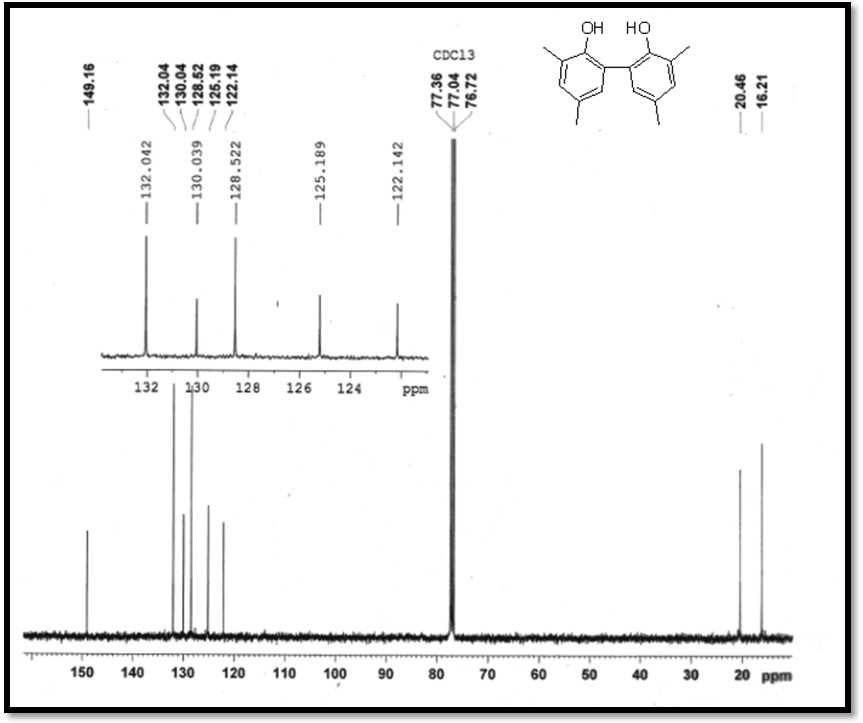
**

**SI-12 ^1^H NMR of 5aa in DMSO-D_6_**

**
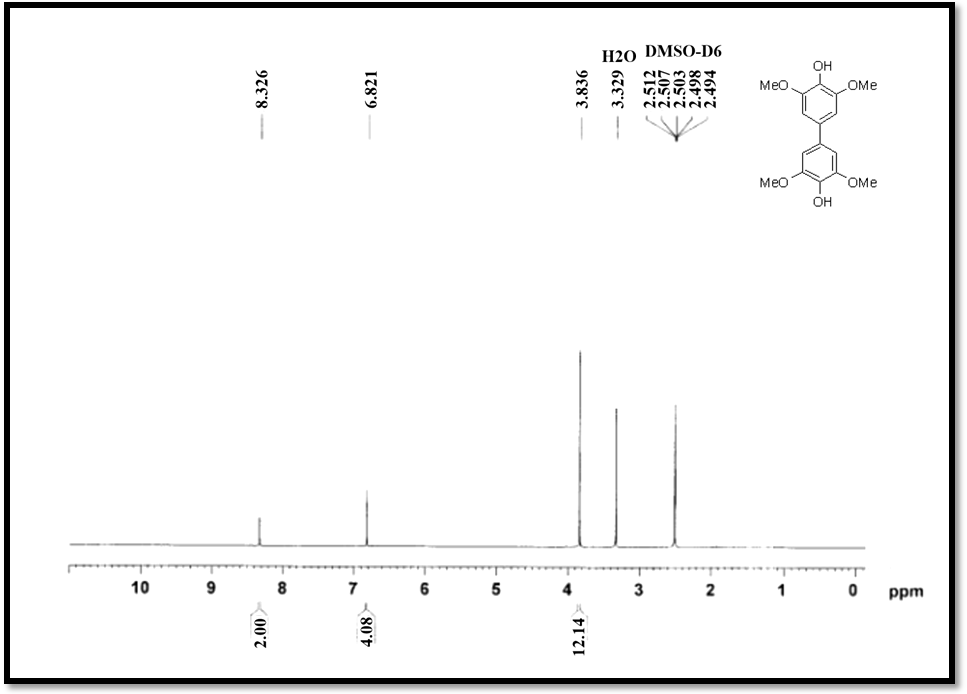
**

**SI-13 ^13^C NMR of 5aa in DMSO-D_6_**


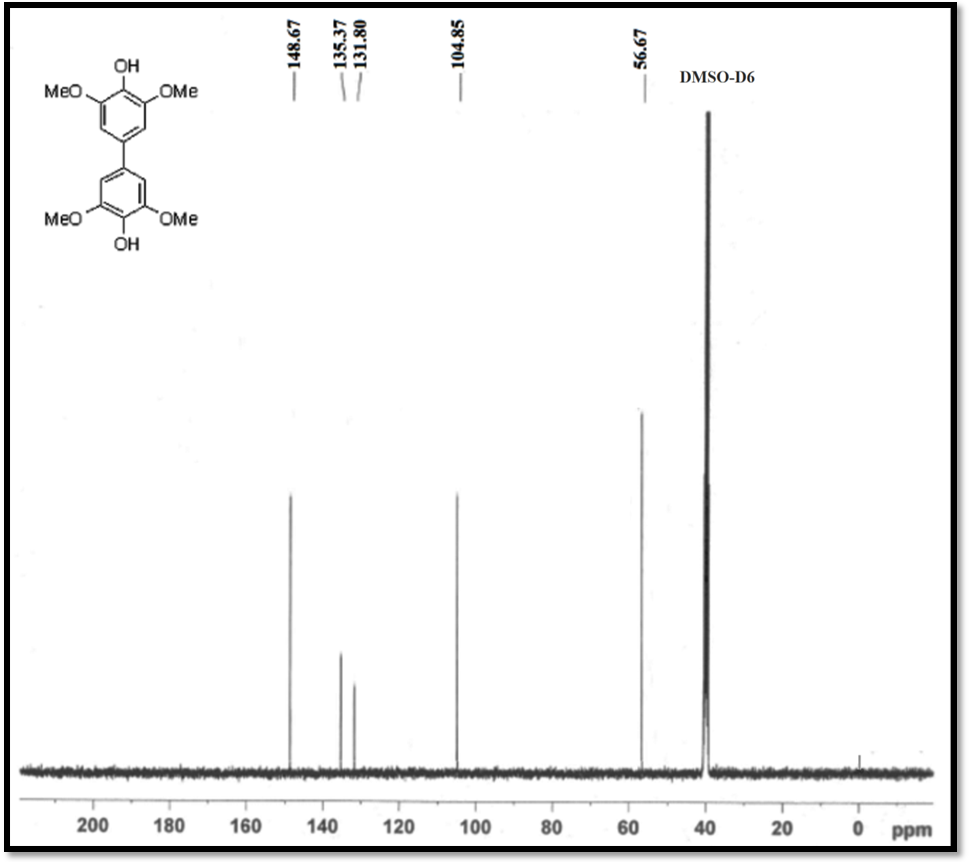


**SI-14 ^1^H NMR of 5bb in CDCl_3_**

**
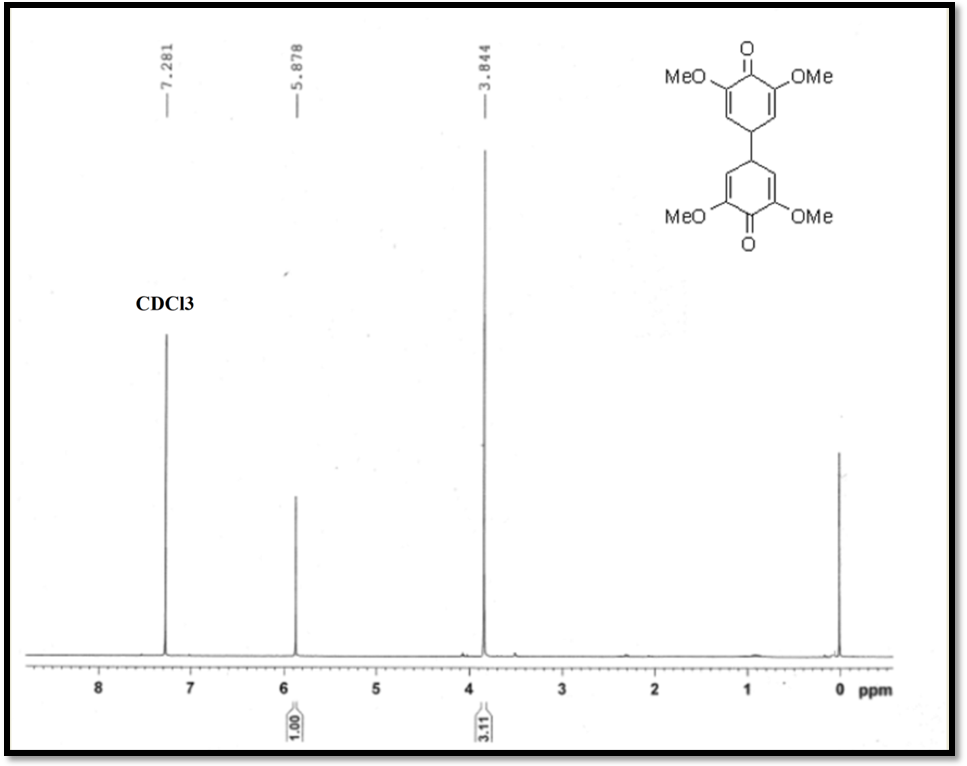
**

**SI-15 ^13^C NMR of 5bb in CDCl_3_**


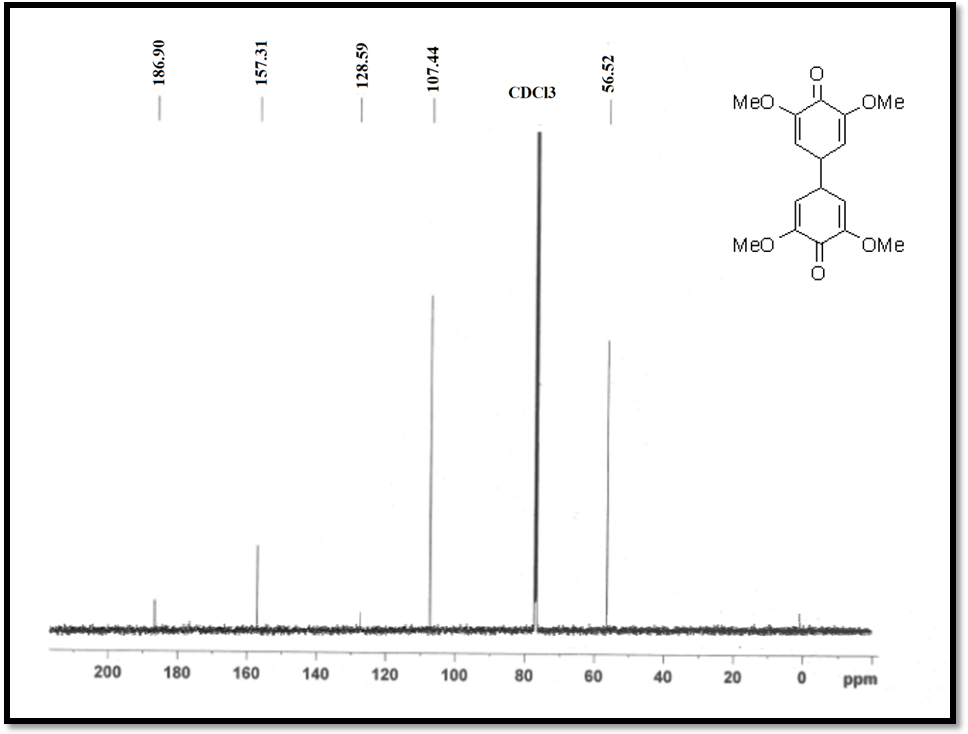


**SI-16 ^1^H NMR of 6aa in CDCl_3_**


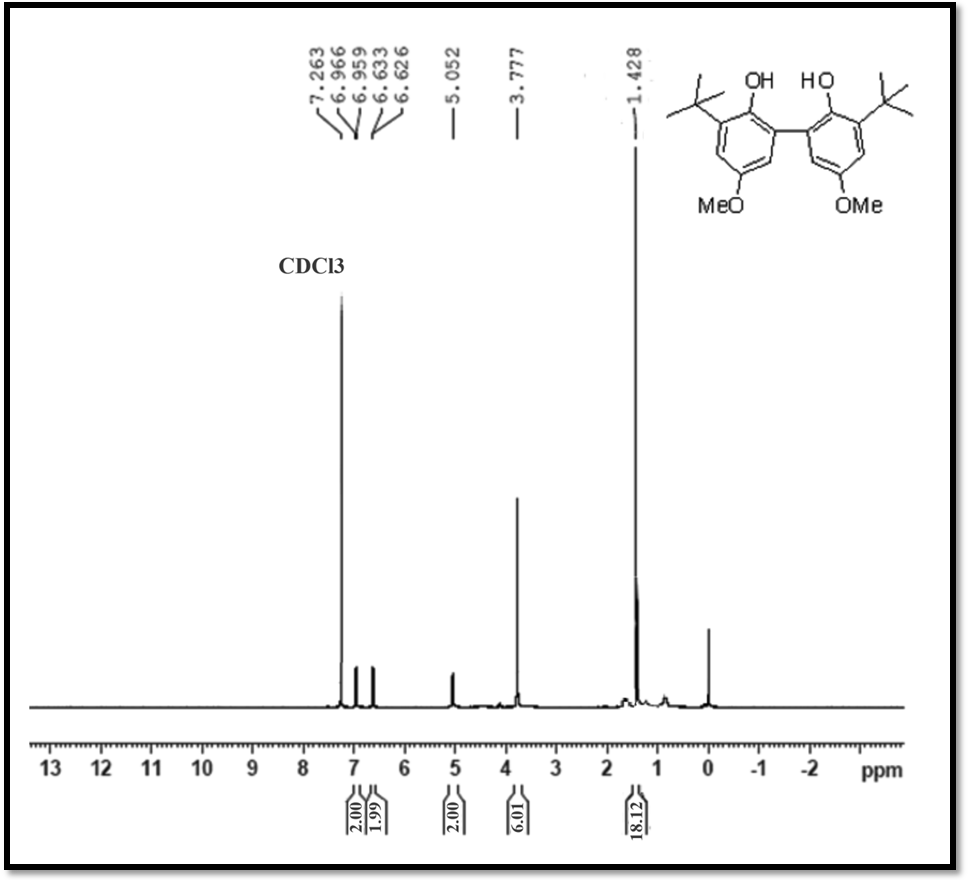


**SI-17 ^13^C NMR of 6aa in CDCl_3_**


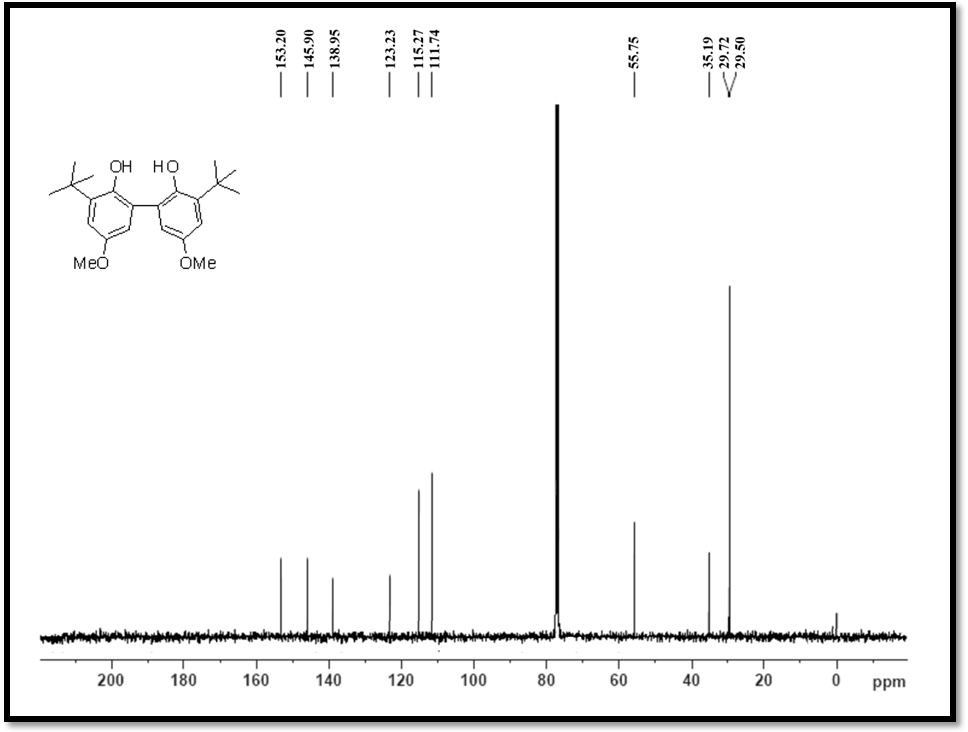


**SI-18 ^1^H NMR of 7aa in DMSO-D_6_**

**
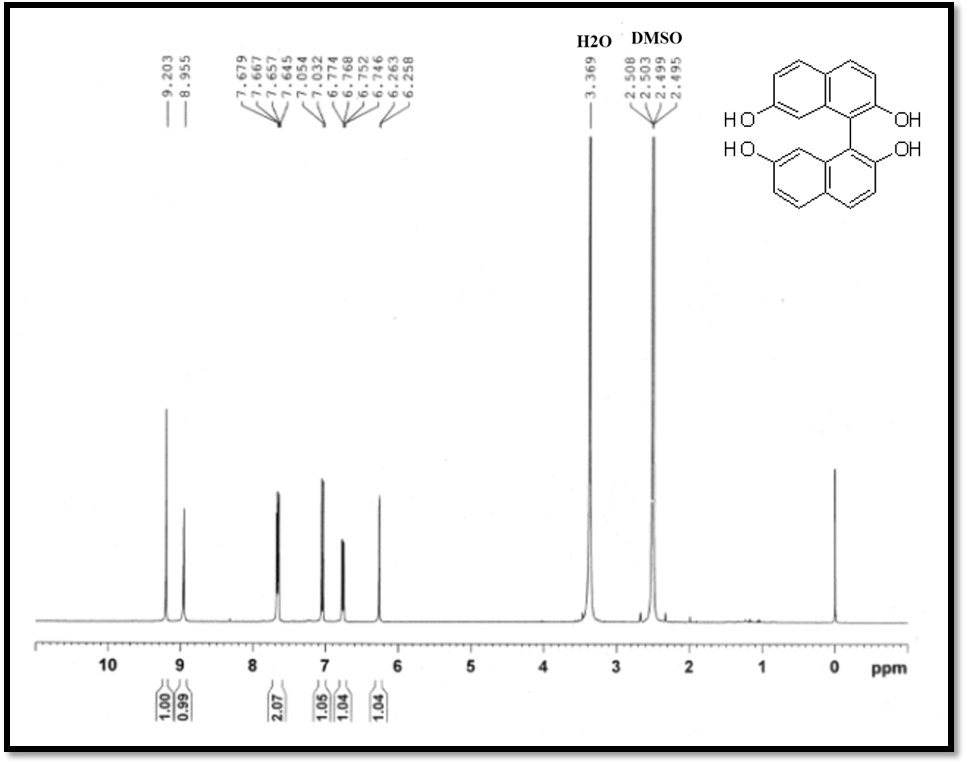
**

**SI-19 ^13^C NMR of 7aa in CDCl_3_**

**
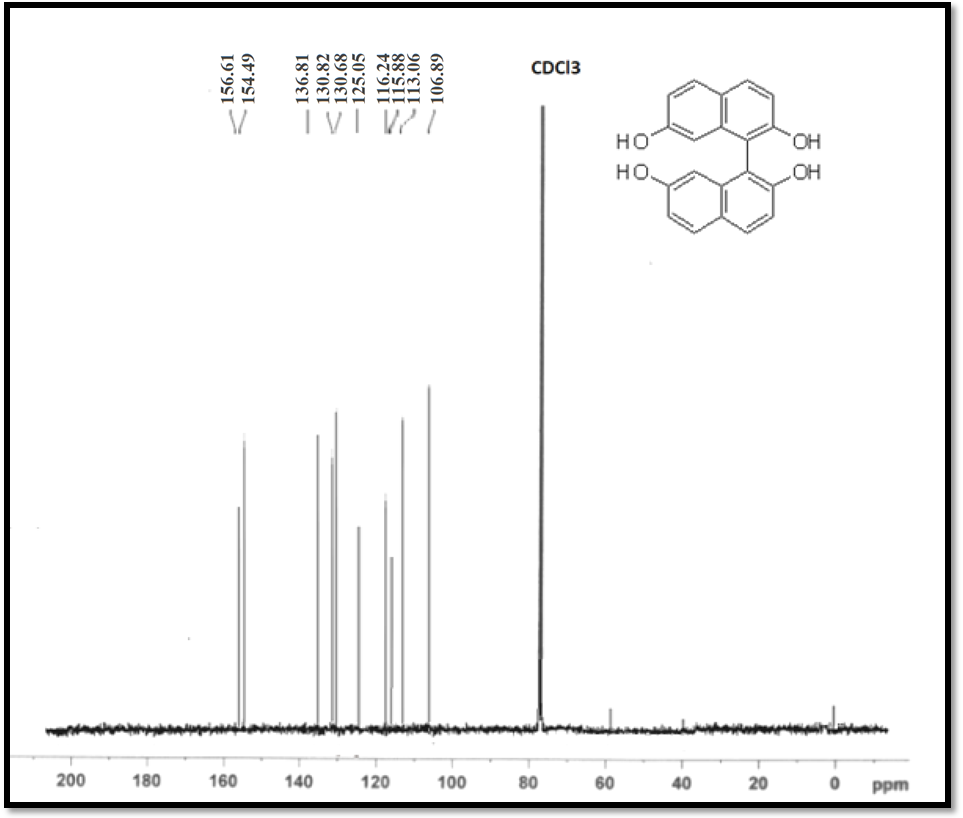
**

**SI-20 ^1^H NMR of c in CDCl_3_**

**
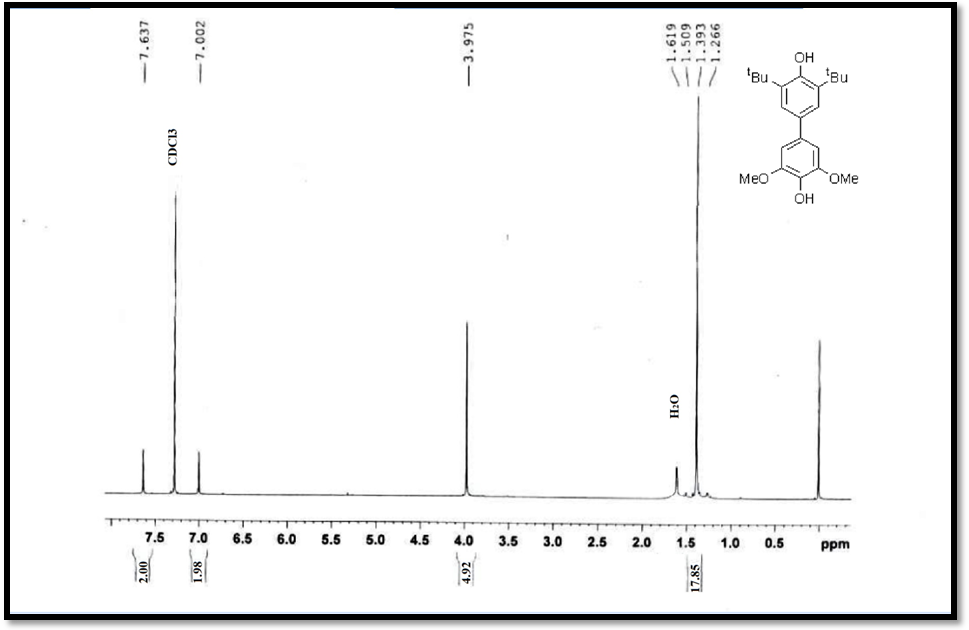
**

**SI-21 ^13^C NMR of c in CDCl_3_**


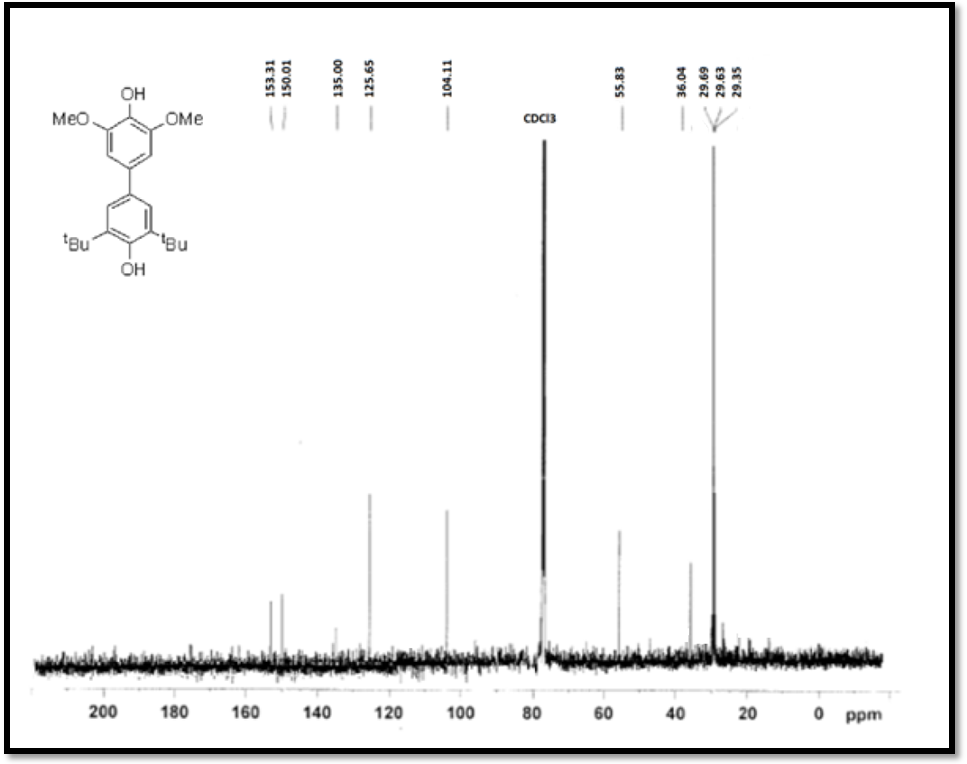


**SI-22 GCMS-1aa**


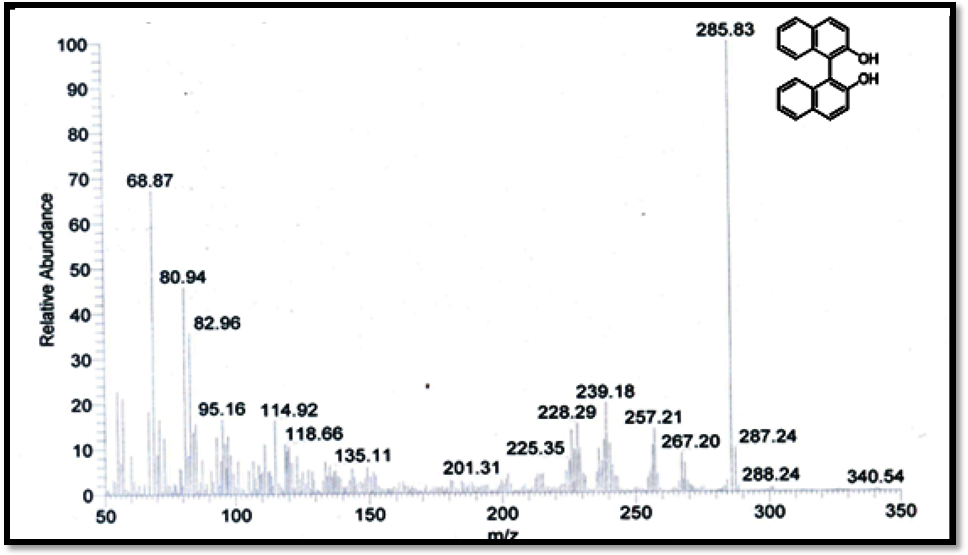


**SI-23 GC-MS of 2aa**


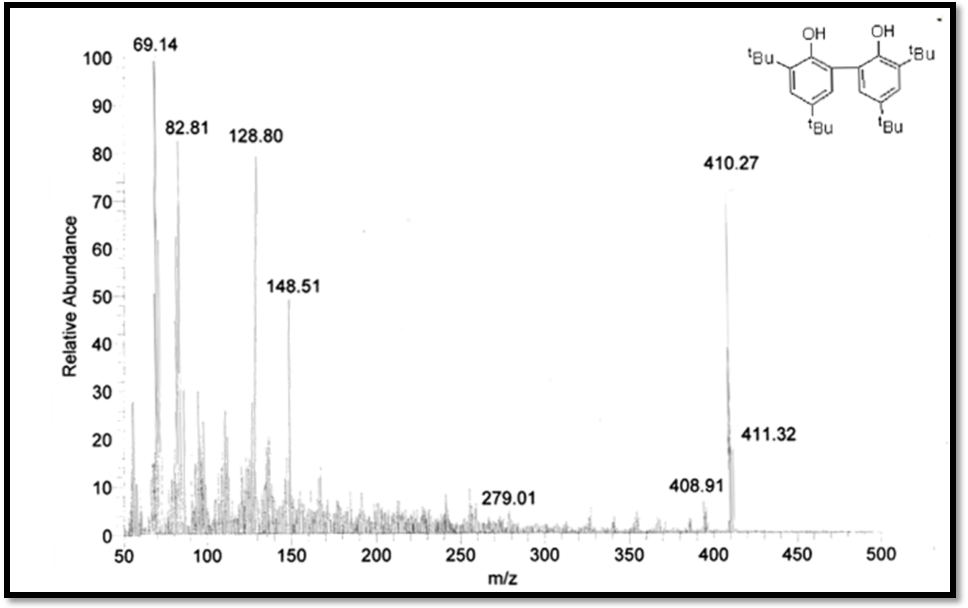


**SI-24 ESI-MS of 3aa**


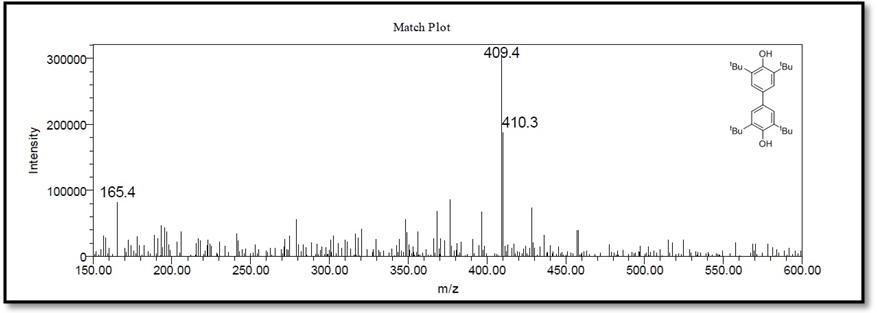


**SI-25 ESI-MS of 3bb**


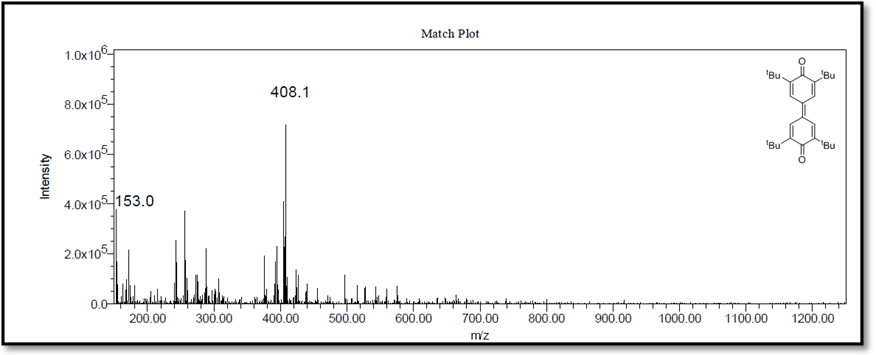


**SI-26 ESI-MS of 4aa**


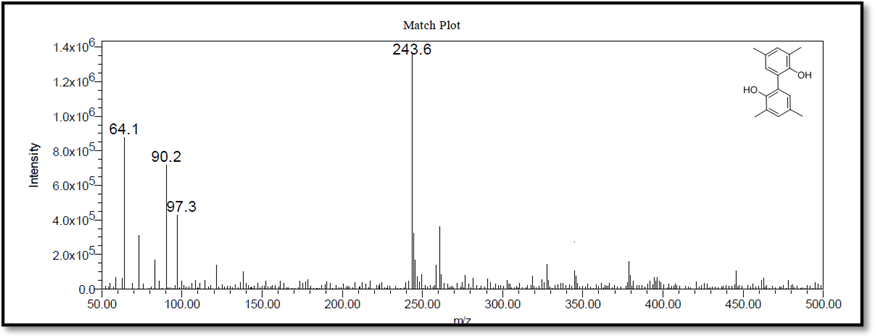


**SI-27 ESI-MS of 5aa**


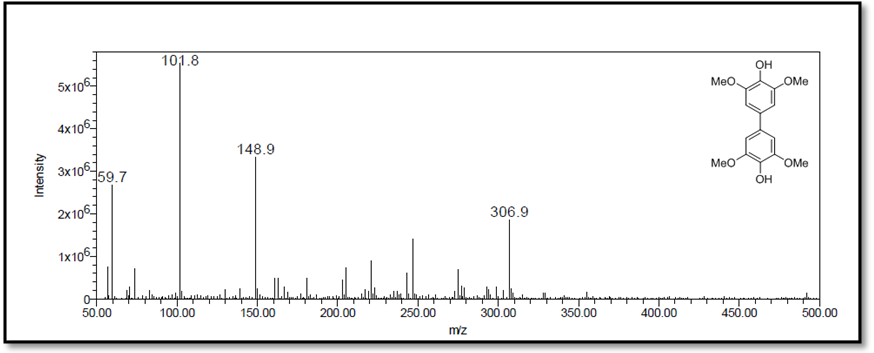


**SI-28 ESI-MS of 5bb**


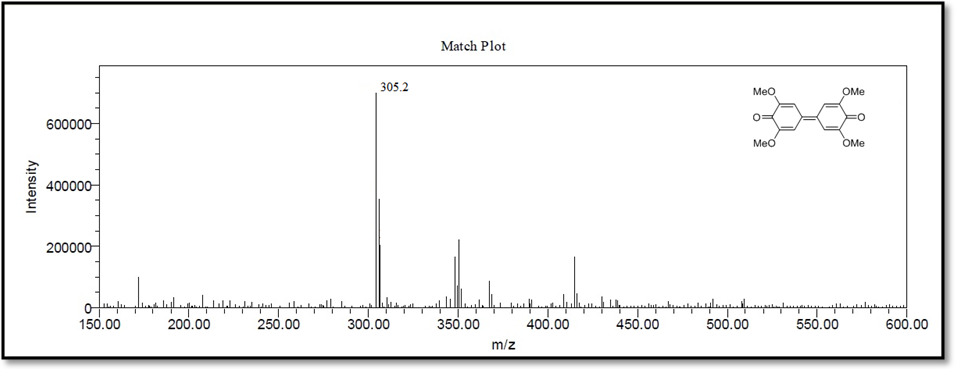


**SI-29 GC-MS of 6aa**


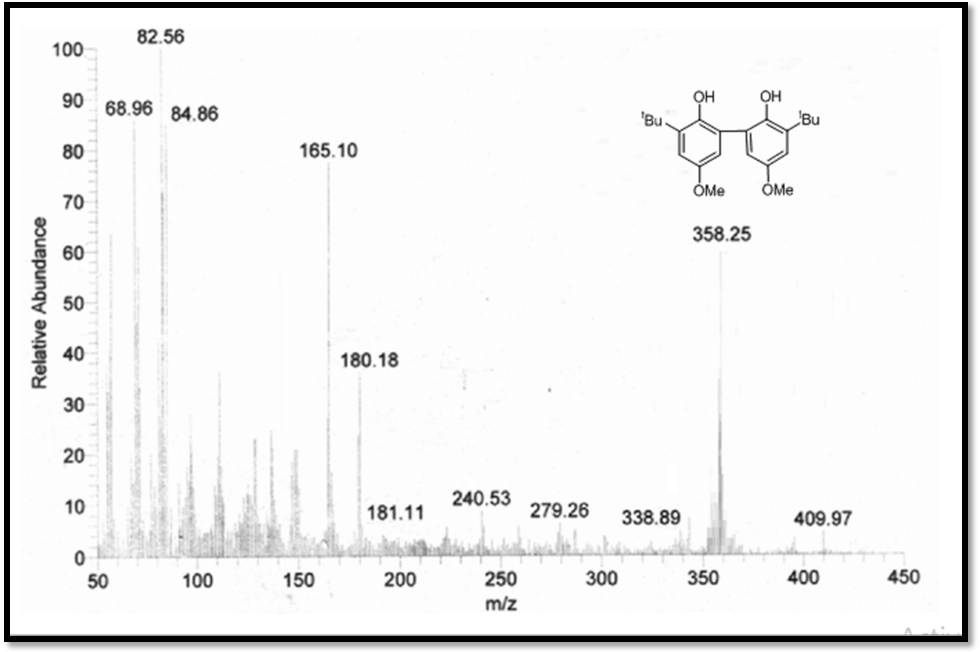


**SI-30 GC-MS of 7aa**


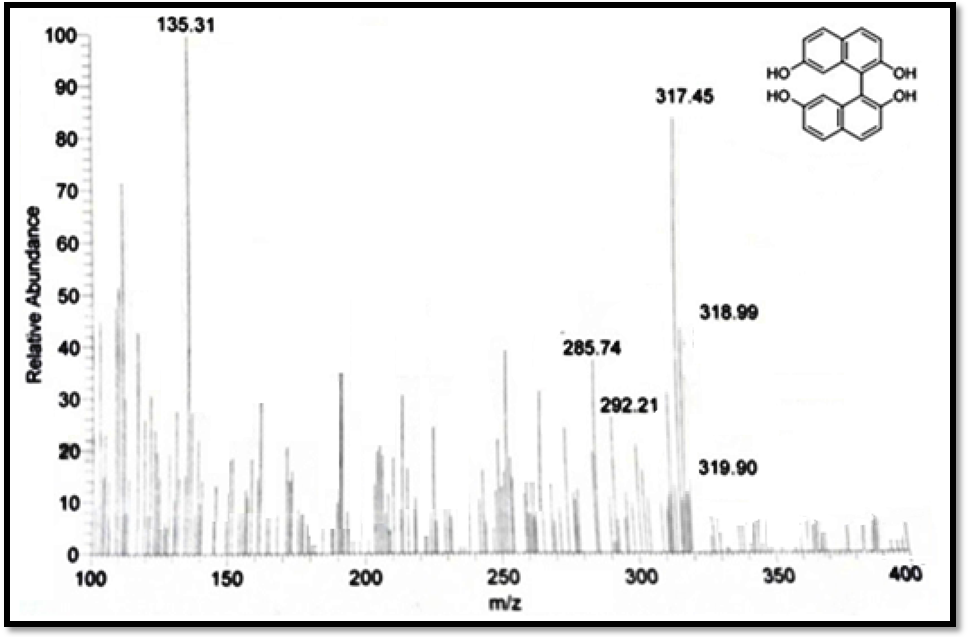


**SI-31 ESI-MS of c**


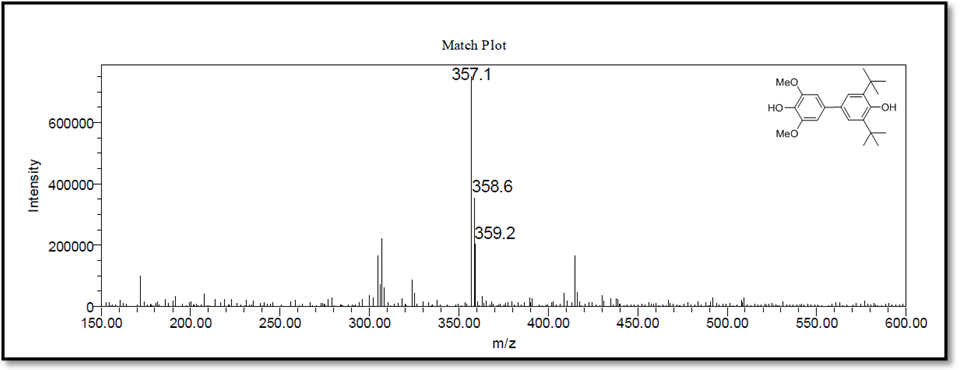


**SI-32 Cross coupling reaction by varying size of vessel surface**

Cross coupling reaction was carried out on polypropylene, durasil glass and Borosilicate glass surface containing vessels as shown in table using general procedure given for experiment 1.

Table presents Cross coupling in different surfaces for complete conversion, and % of isolated product. Range c: range is given for cross coupling products formed in the total yield in repeated experiments

| No. | Surface area in cm^2^ | Surface | Cross coupled  product in% c | Homo coupled products in % | |
| --- | --- | --- | --- | --- | --- |
|  |  |  |  | **1cc** | **1ee** |
|  |  | **Borosilicate glass** | | | |
| 1 | 8 | 5.0 ml test-tube | 34 | 55 | 52 |
| 2 | 8 | 10.0 ml test tube | 35 | 56 | 55 |
| 3 | 10 | 5.0 ml beaker | 29 | 60 | 58 |
| 4 | 10 | 10.0 ml beaker | 29 | 63 | 58 |
| 5 | 12 | 25.0 ml beaker | 31 | 58 | 57 |
|  |  | **Plastic Polypropylene** | | | |
| 6 | 8 | 5.0 ml beaker | 58 | 26 | 23 |
| 7 | 10 | 10.0 ml beaker | 63 | 22 | 22 |
| 8 | 12 | 25.0 ml beaker | 65 | 23 | 25 |
| 9 | 12 | 50.0 ml beaker | 60 | 25 | 22 |
|  |  | **Durasil glass** | | | |
| 10 | 8 | 5.0 ml test-tube | 48 | 44 | 35 |
| 11 | 8 | 10.0 ml test tube | 50 | 42 | 32 |
| 12 | 10 | 5.0 ml beaker | 52 | 40 | 36 |
| 13 | 10 | 10.0 ml beaker | 50 | 42 | 38 |
| 14 | 12 | 25.0 ml beaker | 53 | 42 | 36 |

Results show that there is no effect of surface area on product selectivity.
